# The perioperative sensory–cognitive interface: multimodal neuroimaging of hierarchical network reconfiguration in perioperative neurocognitive disorders

**DOI:** 10.3389/fnins.2026.1956385

**Published:** 2026-10-21

**Authors:** Nanbo Luo, Zhengjun Liu, Zhiheng Liu

**Affiliations:** 1 Department of Anesthesiology, Institute of Translational Medicine, Shenzhen Second People’s Hospital/The First Affiliated Hospital of Shenzhen University, Shenzhen, China; 2 Department of Anesthesiology, The First Affiliated Hospital of Jinan University, Guangzhou, China

**Keywords:** anesthesia, brain networks, multimodal neuroimaging, perioperative neurocognitive disorders, postoperative delirium, sensory–cognitive interface, structure–function coupling, thalamocortical connectivity

## Abstract

Perioperative neurocognitive disorders (PNDs) are commonly attributed to neuroinflammation, cerebrovascular stress, anesthetic neurophysiology, and pre-existing brain vulnerability. These mechanisms identify important insults but do not fully explain how they become disturbances of attention, perception, memory, executive control, and fluctuating awareness. We examine whether changes at the sensory–cognitive interface help explain these symptoms, focusing on postoperative delirium (POD) and early recovery within the broader PND classification. The interface comprises distributed, bidirectional operations that transform sensory evidence into context-sensitive representations through thalamocortical routing, integration, attention, and association systems. The framework makes three testable predictions: sensory function should add explanatory value beyond frailty and brain burden; perioperative effects should vary across hierarchical levels and time rather than reflect uniform suppression; and within-person recovery trajectories should explain outcome better than isolated scans. Anesthetic state-transition studies show that primary sensory responses can persist while feedback, thalamic broadcasting, and distributed integration are disrupted, but whether these changes cause PND remains unproven. Direct perioperative MRI and EEG studies identify pre-existing dysconnectivity, altered sensory and association-network organization, abnormal slow-wave and auditory-feedback dynamics, and incomplete postoperative recovery; nevertheless, findings are heterogeneous and sensory-specific tasks remain uncommon. These observations are compatible with established vulnerability–perturbation models and suggest that symptoms may depend on which networks recover and when. Structural–functional connectivity (SC–FC) coupling is one candidate measurement, not an established biomarker or a requirement of the framework. Longitudinal studies integrating sensory phenotyping, domain-specific cognition, delirium surveillance, MRI, EEG, physiology, and molecular markers should test whether this framework adds reproducible value beyond vascular injury, diffuse arousal failure, and latent neurodegeneration.

## 1 Introduction

The 2018 consensus nomenclature placed pre-existing neurocognitive disorder, postoperative delirium (POD), delayed neurocognitive recovery within 30 days, and postoperative neurocognitive disorder up to 12 months under the umbrella of perioperative neurocognitive disorders (PNDs) (Evered et al., 2018). This framework was designed to align perioperative research with contemporary neurocognitive classification, not to imply that the included syndromes share a single mechanism. POD is an acute, fluctuating disorder of attention and awareness; delayed neurocognitive recovery and postoperative neurocognitive disorder require objective decline on neuropsychological testing, contextualized by subjective concern and functional impact. Emergence agitation, residual sedation, sickness behavior, and transient psychomotor slowing may overlap clinically but should not be used interchangeably. Table 1 separates these phenotypes, time windows, ascertainment requirements, and major mimics.

**Table 1:** Terminology and clinical phenotypes relevant to perioperative cognitive research.

Phenotype	Time window	Core definition	Minimum ascertainment	Primary neural question	Major mimic/confound
Pre-existing neurocognitive disorder	Before surgery	Objective cognitive impairment already present; severity depends on functional independence	Baseline cognitive battery plus functional history; collateral information when needed	What reserve and pathology exist before exposure?	Low education, depression, sensory loss, fatigue, language or cultural bias
Immediate emergence phenomena	Minutes to hours	Delayed emergence, emergence agitation, or residual sedation near anesthetic offset	Arousal and behavior with drug, temperature, metabolic, ventilation, and pain context	How does the brain exit the anesthetized state?	Residual drug, hypothermia, hypercapnia, hypoglycemia, pain, airway distress
Postoperative delirium (POD)	Hours to days; fluctuating	Acute disturbance of attention and awareness with additional cognitive change and fluctuating course	Repeated validated bedside assessment plus chart review; severity and motor subtype recorded	Which arousal, attention, feedback, and contextual-integration processes fail?	Residual sedation, dementia without acute change, aphasia, psychosis, sleep, sickness behavior
Delayed neurocognitive recovery	Up to 30 days	Objective postoperative cognitive decline relative to baseline under the 2018 nomenclature	Domain-based testing with reliable-change or normative method and subjective/functional context	Which processes recover slowly after acute exposure?	Practice effects, pain, sleep loss, medication, mood, intercurrent illness
Postoperative neurocognitive disorder	30 days to 12 months	Mild or major NCD diagnosed postoperatively with objective decline and functional context	Baseline and follow-up neuropsychology; subjective concern; functional assessment	Is persistent dysfunction due to failed recovery, direct injury, or unmasked disease?	Aging, incident neurological disease, preclinical neurodegeneration, non-surgical illness
Subjective cognitive concern	Any postoperative time	Patient, informant, or clinician perceives decline	Structured report linked to daily function; not sufficient alone for objective PND	Does network change have lived functional relevance?	Mood, expectation, fatigue, sleep, caregiver stress
Screening abnormality	Any time	Abnormal score on a brief global or delirium screen	Appropriate cut-off and sensory/language adaptation; confirmatory assessment where required	Can a rapid clinical signal guide further evaluation?	Sensory impairment, education, motor limitation, residual medication
Sickness behavior/postoperative slowing	Hours to days	Reduced motivation, psychomotor speed, appetite, and activity during systemic recovery	Clinical context and repeated examination; absence of defining delirium features	How much cognitive change is nonspecific state effect?	Inflammation, anemia, infection, opioids, immobility

Phenotypes are separated by timing, defining features, and method of ascertainment. They should not be pooled without an explicit rationale.

Early international cohort studies established that older patients can show measurable cognitive decline after both cardiac and non-cardiac surgery and that a vulnerable subgroup has persistent impairment (Moller et al., 1998; Monk et al., 2008; Steinmetz et al., 2009). A recent meta-analysis of older adults undergoing non-cardiac surgery estimated that postoperative cognitive dysfunction was most common during the first postoperative weeks and declined markedly over subsequent months, while also documenting pronounced methodological heterogeneity (Huang et al., 2025). POD is itself associated with worse functional recovery and a higher long-term risk of cognitive decline or dementia, although residual confounding and reverse causation remain important because delirium may expose pre-existing neurodegenerative vulnerability rather than independently cause all subsequent decline (Saczynski et al., 2012; Goldberg et al., 2020; Fong and Inouye, 2022). Contemporary clinical guidance therefore treats PND as a family of interacting phenotypes embedded in patient vulnerability, procedural stress, and recovery context rather than as a unitary “anesthetic toxicity” syndrome (Dilmen et al., 2024; Wu et al., 2025; Zhou et al., 2026).

Most mechanistic accounts begin with plausible upstream perturbations: systemic and central inflammation, blood–brain barrier disruption, endothelial and microvascular dysfunction, cerebral hypoperfusion, oxidative stress, neurotransmitter imbalance, disturbed sleep, pain, and drug effects (Wilson et al., 2020; Devinney et al., 2023). How these processes disrupt communication between brain regions, leading to impaired attention and perception, remains incompletely understood.

We use the term sensory–cognitive interface for that architecture. It is not a new anatomical organ, a diagnostic label, or a replacement for inflammatory and vascular mechanisms. It is an operational systems-level construct encompassing the distributed processes that encode sensory evidence, regulate its gain, integrate information across modalities and time, compare input with prior expectations, assign salience, select information for attention, and make representations available to memory, executive control, and conscious report. The interface is intrinsically bidirectional: cognition is constrained by afferent information, while prior knowledge, goals, and internal state shape early sensory processing through feedback.

To prevent this construct from becoming an omnibus label, we impose an operational boundary. A finding supports the interface account only when it links at least two of three measurable domains: sensory evidence fidelity (including exteroception, interoception, and nociception), hierarchical routing or contextualization, and domain-specific cognitive or behavioral expression. A global change in arousal, inflammation, or connectivity without sensory-to-cognitive specificity is compatible with several competing models and is not sufficient evidence for this framework.

We review anesthesia-state, aging, and direct perioperative studies, distinguishing what each can establish. The principal question is whether impaired use of sensory evidence helps explain attention and perceptual disturbance in POD beyond global arousal failure. We also consider how far this explanation extends to later cognitive outcomes, without assuming a shared mechanism. We define the interface through links among sensory evidence fidelity, hierarchical routing or contextualization, and domain-specific cognitive expression. We then compare the evidence categories, including null findings and studies reporting changes in opposite directions. This comparison informs falsifiable longitudinal predictions based on sensory phenotyping and recovery trajectories, with SC–FC coupling as one candidate measurement. Throughout, we distinguish observations from anesthetized volunteers or experimental animals from direct patient evidence, and established findings from testable hypotheses.

## 2 Literature identification and evidential framework

This is a critical narrative Review rather than a systematic review or meta-analysis. During revision on 21 September 2026, five targeted search modules were executed in PubMed and Europe PMC, covering POD with sensory processing/EEG, POD with MRI/networks, human intracranial anesthetic evidence, ketamine/esketamine in older surgical patients, and EEG-guided anesthetic depth. Exact search strings, date limits, database return counts, and narrative selection principles are provided in the Supplementary Methods. We prioritized peer-reviewed human evidence, longitudinal perioperative cohorts, studies with validated cognitive or delirium ascertainment, major randomized trials, and foundational work needed to define the proposed architecture. Animal studies were retained only when they addressed a mechanistic step that is difficult to interrogate in humans. Publications were selected for conceptual relevance and evidential diversity, including null and directionally discordant findings; the search was not used to estimate pooled prevalence or effect size. The perioperative evidence map prioritizes original human perioperative MRI or EEG studies with defined clinical outcomes and measurement timing; nonsurgical anesthesia experiments, animal studies, reviews, protocols, and purely behavioral trials were not classified as direct perioperative imaging evidence. Related publications do not necessarily represent independent cohorts. Selection was narrative and question-driven; not all returned records underwent eligibility screening.

We group the evidence into four categories according to its relevance to the proposed mechanism; the categories do not rank study quality. Direct perioperative evidence comprises measurements obtained before, during, or after surgery and linked to delirium or cognitive outcomes. Perturbational evidence comprises human anesthesia studies that identify reversible changes in sensory, thalamic, electrophysiological, or large-scale network organization but do not establish PND mechanisms. Vulnerability evidence derives mainly from aging, sensory loss, cerebrovascular disease, and cognitive-reserve research. Mechanistic bridge evidence includes cellular or animal work connecting surgical inflammation and physiological stress to brain dysfunction. These categories are complementary but not interchangeable. We did not register a protocol, conduct dual independent screening, calculate pooled imaging effects, or perform formal study-level risk-of-bias scoring. Consequently, completeness cannot be assumed and mechanistic claims are weighted qualitatively according to design, temporality, replication, sample size, phenotype quality, and control of motion, physiology, medication, and attrition. The model should be evaluated against direct evidence, competing explanations, and the specificity of its prospective predictions.

## 3 Operational definition and neurobiological architecture of the sensory–cognitive interface

Figure 1 summarizes the proposed hierarchy and its bidirectional information flow; Table 2 translates each level into candidate perioperative failures, measurements, and current evidence status.

**Figure 1:**
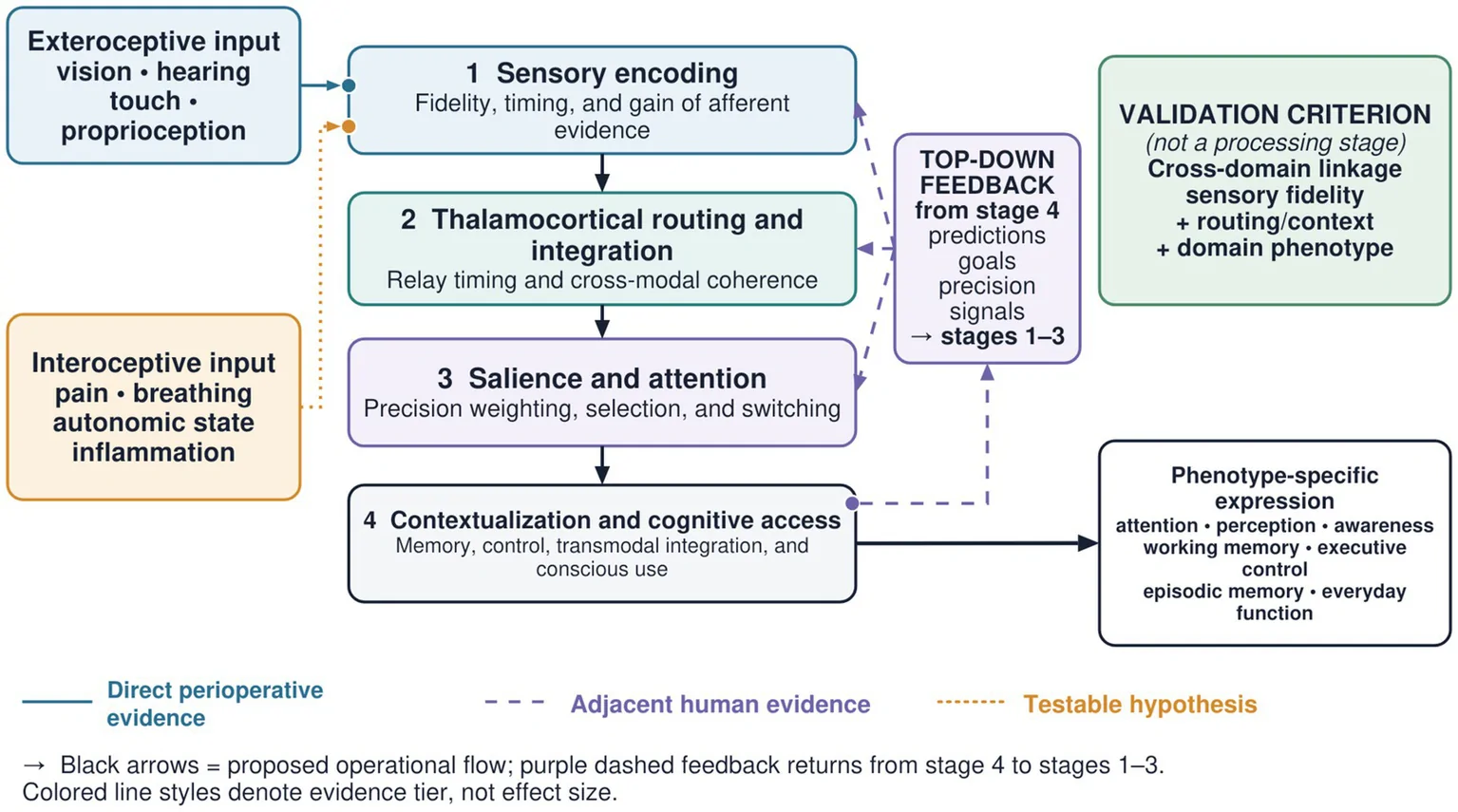
Operational architecture and evidence boundary of the perioperative sensory–cognitive interface. Bottom-up exteroceptive and interoceptive evidence is routed through thalamocortical systems, integrated across modalities, selected by salience and attention, and contextualized by memory, control, and transmodal systems. Top-down predictions, goals, and precision signals modulate each level. Black arrows denote proposed operational information flow and cross-domain linkage; colored solid, dashed, and dotted paths distinguish direct perioperative evidence, adjacent human evidence, and testable hypotheses. A finding supports the interface account only when it links sensory evidence, hierarchical routing or contextualization, and cognitive expression. The evidence labels apply to individual links; the full pathway remains a hypothesis.

**Table 2:** Operational levels of the sensory–cognitive interface and candidate measurements.

Operational level	Representative systems	Normal operation	Candidate perioperative failure	Candidate measures	Current evidence status
Exteroceptive encoding	Visual, auditory, somatosensory cortex; modality-specific thalamus	Encode stimulus features and timing	Degraded input, anesthetic suppression, sensory-aid removal	Acuity / speech-in-noise; evoked EEG; task fMRI; sensory-network FC	Direct perioperative evidence limited but growing
Interoception and nociception	Insula, anterior cingulate, brainstem, somatosensory systems	Represent bodily state and action relevance	Pain, dyspnea, nausea, inflammation dominate salience	Pain ratings; autonomic measures; insula/salience FC; evoked potentials	Strong adjacent evidence; sparse perioperative mediation
Thalamocortical routing	Core–matrix thalamus; basal forebrain; ascending arousal systems	Relay, broadcast, synchronize, and gate by state	Weak matrix-to-transmodal broadcast; unstable arousal	Thalamic gradients; thalamocortical FC; EEG spectra/complexity	Strong anesthesia-state evidence; direct PND evidence emerging
Multisensory integration	Superior temporal, posterior parietal, insular association cortex	Bind congruent evidence across modality and time	Widened binding window; conflict, deprivation, or overload	Audiovisual tasks; congruence gain; task fMRI; EEG mismatch	Aging evidence strong; perioperative evidence sparse
Predictive feedback	Hierarchical corticocortical and transthalamic loops	Compare priors with evidence; weight precision	Feedback loss; unreliable precision; prediction-error instability	Mismatch negativity; dynamic causal modeling; laminar/invasive EEG	Task-based POD auditory-feedback evidence; DCM inference needs independent replication
Salience and attention	Salience/VAN, DAN, cingulo-opercular systems	Detect relevance, reorient, maintain selection	Unstable switching; pain/interoceptive capture; poor sustained attention	Within/between-network FC; task reorienting; EEG vigilance	Repeated POD associations across modalities
Executive access	Frontoparietal control and transmodal association cortex	Maintain goals; integrate information for flexible report	Restricted long-range integration and conscious access	Network segregation; integration; gradient position; working-memory task	Strong anesthesia evidence; heterogeneous perioperative evidence
Memory binding	Hippocampus, medial temporal, DMN	Bind context across time and support retrieval	Impoverished encoding, weak hippocampal–DMN coordination	Domain memory scores; hippocampal/DMN FC; task fMRI	Direct studies present; specificity remains uncertain
Structure–function translation	White-matter connectome plus functional networks	Balance structural efficiency and functional flexibility	Spatially inappropriate recoupling or decoupling during recovery	Regional/edge/network SC–FC coupling; graph-signal decoupling	Perioperative biomarker hypothesis—not yet validated

The interface is a bidirectional hierarchy, not a single network. Each level supports distinct, testable operations. Directions are network-, region-, method-, and time-specific.

### 3.1 From distributed processing to a cortical hierarchy

Classical models already rejected a strict separation between sensory and associative cortex. Mesulam (1998) described cognition as distributed processing across transmodal, heteromodal, and modality-specific zones rather than a serial progression through isolated centers. Contemporary connectomics has made this architecture measurable. The principal cortical gradient places visual and somatomotor regions at one extreme and default-mode, temporoparietal, and medial prefrontal regions at the other, with attention and control networks occupying intermediate positions (Margulies et al., 2016). Convergent analyses of anatomical connectivity, microstructure, laminar differentiation, and functional connectivity support a stepwise transition from highly differentiated sensory systems to integrative transmodal cortex, while also showing that structural and functional hierarchies are related but not identical (Sepulcre et al., 2012; Huntenburg et al., 2018; Paquola et al., 2019).

Primary sensory regions have short temporal receptive windows, dense local circuitry, and relatively direct thalamic drive. Transmodal regions integrate information over longer timescales and larger spatial contexts, support counterfactual and internally generated representations, and depend more strongly on distributed recurrent interactions (Hasson et al., 2008; Murray et al., 2014). A patient can therefore retain cortical responses to sound or touch yet fail to integrate them with place, time, goals, and memory.

### 3.2 Thalamic gating, core–matrix organization, and arousal

The thalamus is central to this architecture but should not be reduced to a passive relay. First-order and higher-order nuclei participate in transthalamic corticocortical communication, regulate cortical synchrony, and route information according to behavioral state (Sherman, 2007). The core–matrix distinction provides one useful, if simplified, organizing principle. Core cells project relatively focally to middle cortical layers and support modality-specific signaling, whereas matrix cells have more diffuse projections to superficial layers and are positioned to coordinate distributed cortical activity (Jones, 2001). Modern anatomical and functional data show gradients rather than a strict binary division, yet the distinction remains helpful when interpreting why anesthesia might preserve some sensory relay while disrupting broad transmodal coordination (Huang et al., 2024; Zhou et al., 2025). Human intracranial recordings provide complementary evidence: propofol reduced thalamocortical alpha/low-beta coherence in 10 patients undergoing deep-brain stimulation (Malekmohammadi et al., 2019), while recordings in 11 epilepsy patients identified distinct sensory and cognitive cortical alpha networks linked to thalamic projections by tractography in matched healthy surrogates (Weiner et al., 2023). Neither study directly identified core and matrix cell types, and these specialized cohorts do not establish the mechanism of POD.

Thalamic function is inseparable from ascending arousal and neuromodulatory systems. Cholinergic and monoaminergic pathways contribute to arousal and cortical state during wakefulness, sleep, and anesthesia (Brown et al., 2010). The sensory–cognitive interface therefore depends on both network topology and physiological operating state: the same anatomical connections can support different information flow under different neuromodulatory regimes.

### 3.3 Multisensory integration and temporal binding

Perception is normally multisensory. Superior temporal, posterior parietal, insular, and frontal regions integrate visual, auditory, somatosensory, vestibular, proprioceptive, interoceptive, and nociceptive evidence. Integration is most beneficial when cues are temporally and spatially congruent and individually uncertain; incongruent or excessive signals instead generate conflict and attentional load (Ernst and Banks, 2002; Stein and Stanford, 2008). The postoperative environment is rich in such conflicts: an unfamiliar room, reduced vision without spectacles, muffled speech without hearing aids, pain and visceral signals, intermittent alarms, nocturnal illumination, and disrupted proprioceptive input during immobility. These factors are not sufficient causes of PND, but they can raise the computational demand on an already perturbed system.

Interoception deserves particular emphasis. The insula, anterior cingulate, brainstem, and somatosensory systems integrate the physiological state of the body with affective salience and action. Pain, dyspnea, nausea, inflammation, and autonomic instability can therefore dominate limited attentional resources and alter the weighting of external sensory evidence (Critchley et al., 2004). In delirium, misperceptions and hallucinations may reflect not simply abnormal external sensing but an unstable balance between external evidence, internal bodily signals, and prior beliefs.

### 3.4 Predictive processing, precision, and conscious access

Predictive-processing accounts propose that cortical hierarchies continuously compare top-down predictions with bottom-up signals, transmitting residual prediction error while adjusting the precision—or reliability—assigned to each source (Rao and Ballard, 1999; Friston, 2005; Friston and Kiebel, 2009; Friston, 2010). Although no single predictive-coding formulation is universally accepted, its concepts are clinically productive. Sensory impairment lowers the precision of afferent evidence; unfamiliar environments increase uncertainty; pain and inflammation amplify salient internal signals; and anesthesia alters feedback and recurrent interactions. If the system assigns too little precision to reliable input, or too much precision to noisy internal predictions, perception may become unstable and context-inappropriate.

Conscious access also requires information to influence distributed systems beyond the region in which it was initially encoded. Global-workspace and related recurrent-processing theories differ in detail, but they agree that local sensory activation is not equivalent to flexible report, memory access, and goal-directed use (Dehaene and Naccache, 2001; Seth and Bayne, 2022). The interface construct therefore separates three events that are often conflated: encoding of a stimulus, integration and selection of its meaning, and broadcast or access to systems that support sustained cognition.

### 3.5 Large-scale networks as interacting operations

Canonical resting-state networks offer a coarse but useful vocabulary. Dorsal attention systems support goal-directed selection; ventral attention and salience systems detect behaviorally relevant change and reorient processing; frontoparietal control systems maintain task rules and coordinate flexible behavior; default-mode systems integrate memory, semantic context, and internally generated models; and hippocampal–medial temporal circuits bind experience across time. The salience network, particularly anterior insula and dorsal anterior cingulate, has been proposed to coordinate switching between internally and externally oriented states (Seeley et al., 2007; Menon, 2011). Large-scale parcellations consistently identify these systems, although boundaries and labels vary (Power et al., 2011; Yeo et al., 2011).

The interface is not synonymous with the “triple-network model,” nor does every cognitive symptom map one-to-one onto a network. Rather, the networks describe interacting operations. Sustained attention may fail because sensory evidence is weak, salience assignment is unstable, control systems cannot maintain a task set, or internally generated activity intrudes. Episodic memory may fail because encoding was impoverished, hippocampal binding was disrupted, or later retrieval could not access distributed representations. This process-level view argues for domain-specific cognitive phenotyping and against treating a global screening score as a complete neural phenotype.

### 3.6 Structure constrains function—but not uniformly

White-matter pathways constrain which regions can communicate efficiently, yet functional interactions also depend on polysynaptic routes, common inputs, dynamics, and state. SC–FC coupling quantifies the correspondence between structural connectivity and functional connectivity, but there is no single canonical measure. Coupling can be estimated regionally, edge-wise, within networks, across subjects, or using graph-signal approaches that decompose functional activity into components aligned with or decoupled from the structural connectome (Preti and Van De Ville, 2019; Fotiadis et al., 2024). Primary sensory cortex tends to be more tightly coupled to direct structural scaffolding, whereas transmodal cortex exhibits greater functional decoupling, consistent with flexible integration across diverse inputs (Valk et al., 2022).

This spatial heterogeneity is central to our argument. High coupling is not universally beneficial. Tight coupling may support efficient sensory transmission, while an appropriate degree of transmodal decoupling may enable flexible cognition. We hypothesize that excessive decoupling could reflect noisy dynamics, whereas excessive coupling could reflect loss of flexibility. Developmental and aging studies show that coupling changes across the lifespan and relates to cognition, heritable factors, and disease burden (Baum et al., 2020; Gu et al., 2021; Zamani Esfahlani et al., 2022; Liu et al., 2024; Zhang H. et al., 2024). We therefore hypothesize that perioperative vulnerability involves abnormal regional patterns and recovery trajectories of SC–FC coupling along the sensory-to-transmodal hierarchy, which a single global coupling value may not capture. Clinically, a patient might hear and recognize a familiar speaker yet fail to integrate the conversation into the hospital context, or recall isolated events without their temporal sequence. These examples illustrate impaired contextual integration; whether they reflect excessive SC–FC decoupling in POD remains untested.

## 4 Baseline vulnerability: why aging changes the response to perioperative stress

### 4.1 Network segregation, dedifferentiation, and cognitive reserve

With aging, resting-state systems become less segregated: within-network connectivity often declines while between-network interactions become less distinct, and lower segregation relates to poorer memory and executive performance beyond chronological age (Chan et al., 2014; Geerligs et al., 2015). Task activations also become less selective, a phenomenon described as neural dedifferentiation (Koen and Rugg, 2019). Changes in functional gradients suggest that the separation between unimodal and transmodal organization can contract with age, potentially reducing the system’s repertoire of distinct network states (Bethlehem et al., 2020).

Not all broader recruitment is pathological. The HAROLD and compensation-related utilization of neural circuits hypotheses describe increased bilateral or additional recruitment that may maintain performance in older adults (Cabeza, 2002; Cabeza et al., 2002). Preoperative hyperconnectivity may indicate successful compensation, inefficient over-recruitment, or reduced network specificity. Therefore, the direction of a connectivity difference cannot be interpreted without performance, longitudinal change, and network context.

Cognitive reserve further modifies clinical expression. Reserve is not a single imaging measure but a property inferred when individuals sustain function despite age- or disease-related burden (Stern et al., 2020). Education and cognitively stimulating experience are imperfect proxies; network efficiency, flexible recruitment, brain maintenance, and sensory health may be closer mediators.

### 4.2 Sensory impairment as chronic precision loss

Hearing and visual impairment are among the most reproducible clinical vulnerability factors for delirium. Longitudinal population studies also associate hearing loss with accelerated cognitive decline and incident dementia, although social isolation, shared vascular pathology, reverse causation, and measurement bias contribute to the association (Valentijn et al., 2005; Lin et al., 2011; Lin et al., 2013; Loughrey et al., 2018). From an interface perspective, sensory loss is not merely a covariate. Degraded input demands greater attentional effort and reliance on predictions, potentially altering long-term network organization.

Aging can increase behavioral benefit from congruent multisensory cues, especially when unisensory signals are weak, yet can also widen temporal binding windows and impair suppression of irrelevant or incongruent information (Baltes and Lindenberger, 1997; Laurienti et al., 2006; Freiherr et al., 2013; de Dieuleveult et al., 2017). This combination—greater dependence on multisensory redundancy but poorer selection under conflict—may be advantageous in a stable environment and fragile in an unfamiliar postoperative setting.

### 4.3 White-matter disease, vascular burden, and structural reserve

White-matter hyperintensities, lacunes, microinfarcts, and reduced tract integrity can weaken long-range communication and lower the threshold at which physiological stress produces disconnection. Classic delirium models describe a balance between baseline vulnerability and precipitating insult: a highly vulnerable patient may develop delirium after a modest stressor, whereas a resilient patient requires a larger insult (Inouye et al., 1993). Meta-analytic risk-factor work supports this multidimensional interaction, although effect estimates vary by setting and ascertainment (Ahmed et al., 2014).

Structural burden need not produce a focal deficit before surgery. Redundant routes and functional compensation can preserve behavior until anesthesia, inflammation, hypotension, or sleep deprivation reduces the operating margin. This distinction matters for interpreting diffusion MRI. Lower fractional anisotropy or reduced global efficiency is not evidence that surgery caused white-matter injury when measured only preoperatively; rather, it may index latent disconnection risk. Conversely, new diffusion lesions after surgery may represent direct injury with consequences distinct from reversible network reconfiguration (Cavallari et al., 2016; Browndyke et al., 2024).

### 4.4 Preoperative SC–FC configuration as a latent vulnerability phenotype

Healthy aging studies show that SC–FC coupling varies with cognitive performance and small-vessel burden, but the field remains methodologically diverse (Liu et al., 2024; Zhang H. et al., 2024). A plausible perioperative vulnerability phenotype would combine: preserved or impaired structural capacity; baseline functional segregation; regional coupling along the cortical hierarchy; sensory function; and cognitive reserve.

The studies reviewed here have not validated SC–FC coupling as a perioperative biomarker for POD or postoperative neurocognitive disorder. It remains of mechanistic interest because structural networks change relatively slowly over days, while functional networks can reorganize rapidly; whether this difference helps explain perioperative cognitive changes requires testing.

## 5 Anesthesia as a perturbational model—not a surrogate for PND

Across drugs, anesthetic unconsciousness is better described as a reconfiguration of communication than as uniform cortical suppression (Alkire et al., 2008; Brown et al., 2010; Mashour, 2024). Local sensory responses, spontaneous activity, and even some forms of implicit processing may persist, while long-range integration, feedback signaling, complexity, and flexible responsiveness decline. This state literature illuminates candidate operations within the sensory–cognitive interface, but it cannot by itself establish that the same changes cause postoperative disorders. Healthy volunteers typically recover completely, anesthetic concentrations differ from clinical practice, surgery and inflammation are absent, and measures obtained during unresponsiveness may reflect a physiological state rather than injury. Table 3 contrasts agent- and exposure-specific neural patterns, evidential context, limitations, and testable perioperative implications.

**Table 3:** Anesthetic agents and perioperative exposures: network evidence, limitations, and testable implications.

Agent/exposure	Observed neural pattern	Evidence context	Interface interpretation	Key limitation	Testable perioperative implication
Propofol	Reduced within/between-network integration; altered thalamocortical geometry; disrupted top-down alpha–beta and bottom-up gamma routing	Human volunteer fMRI/EEG and invasive recordings	Sensory encoding may persist while feedback, broadcasting, and transmodal access fail	State change is not injury; dose and vascular effects vary	Age-adjusted network recovery, not drug dose alone, should predict cognition
Sevoflurane/volatile agents	DMN, VAN, frontal, and thalamic connectivity decline; primary sensory networks relatively preserved at moderate depth	Human fMRI and simultaneous EEG	Higher-order integration can fail before primary sensory response	BOLD vascular effects; agent and concentration dependence	Measure end-tidal dose, physiology, EEG, and post-anesthetic recovery jointly
Dexmedetomidine	Sleep-like but pharmacologically distinct EEG and network topology; lighter states remain arousable	Human sedation studies; selected clinical trials	May preserve a different route to responsiveness and sleep organization	Not physiological sleep; bradycardia/hypotension; context-dependent clinical effects	Test whether benefit is mediated by sleep, opioid reduction, or network trajectory
Ketamine	Dissociative, drug-specific thalamocortical and frontoparietal reconfiguration; complexity may be preserved or altered	Volunteer imaging plus perioperative trials	Unresponsiveness is not a single disconnection state	Psychotomimetic effects; mixed clinical results across dose, formulation, and surgical context	Avoid universal depth metrics; model agent-specific recovery states
Opioids and analgesia	Pain engages salience/interoceptive networks; opioids reduce nociception but depress arousal	Pain imaging and clinical observational evidence	Both uncontrolled pain and excessive sedation can destabilize the interface	Confounding by indication and surgery	Test opioid-sparing strategies with pain, arousal, EEG, and cognition together
Regional vs general anesthesia	Different acute neural exposures; randomized trials show no universal POD advantage for regional/spinal strategy	RAGA and REGAIN clinical trials	Technique labels conceal sedation, analgesia, hemodynamics, and conversion	Not a drug-free contrast; procedures and cointerventions vary	Quantify achieved exposure and recovery trajectory rather than nominal technique
EEG-guided depth	Can reduce dose and suppression; delirium benefit inconsistent across trials	BIS/depth trials; ENGAGES: no POD reduction; BALANCED: lower POD with BIS 50 vs. 35	EEG may identify susceptibility as well as exposure	Proprietary indices compress age-dependent spectra	Target explicit spectral/suppression phenotypes and test mediation
Hemodynamic stress	Hypoperfusion and vascular BOLD effects may alter function; strategic injury can disconnect networks	Observational studies and individualized-BP trials	Reduced physiological margin can expose latent structural vulnerability	Threshold definitions and confounding differ	Model baseline pressure, duration, autoregulation, ASL, and DWI lesions
Inflammation/BBB dysfunction	Cytokines and microglial/endothelial responses alter synaptic gain, metabolism, and neurovascular coupling	Animal mechanisms plus human biomarkers	May destabilize precision, salience, and network re-segregation	Human causal mediation sparse; biomarkers nonspecific	Align serial inflammatory markers with EEG/fMRI trajectory
Sleep/circadian disruption	Altered slow-wave activity, attention, and DMN–control interactions	Sleep science and perioperative observational studies	Reduces arousal stability and contextual integration during recovery	Bidirectional relationship with delirium	Randomize sleep–circadian bundle and measure network target engagement
Sensory deprivation/overload	Low-fidelity or conflicting input increases attention and prediction demands	Aging, sensory-loss, and multicomponent prevention evidence	Can amplify precision error in a low-reserve network	Direct neural mediation after surgery unproven	Measure sensory function and environment as modifiers, not nuisance variables

State-transition and clinical evidence are distinguished. No exposure has a universally beneficial connectivity direction. Directions are network-, region-, method-, and time-specific.

### 5.1 Propofol: integration, feedback, and thalamocortical geometry

Propofol studies provide some of the clearest evidence for hierarchical disconnection. Resting-state fMRI demonstrates breakdown of both within-network and between-network connectivity during loss of consciousness, with prominent effects in frontoparietal, default-mode, and thalamocortical systems (Boveroux et al., 2010; Stamatakis et al., 2010). Graph analyses suggest reduced integration and altered community structure rather than a simple global reduction in correlation (Schrouff et al., 2011; Schröter et al., 2012). Thalamic and association-cortical changes are substantial, yet primary sensory regions may retain stimulus-evoked responses. The critical deficit is therefore plausibly the capacity of sensory information to propagate, interact with contextual feedback, and influence distributed cognitive systems.

Recent work refines this account at two levels. Functional-gradient mapping indicates that propofol changes thalamocortical organization in a manner that preferentially weakens transmodal aspects of core–matrix architecture (Huang et al., 2024). Intracortical recordings show that propofol-mediated loss of consciousness disrupts predictive routing: top-down alpha–beta interactions and bottom-up gamma-related signaling become uncoupled, impairing reciprocal communication across cortical hierarchy (Xiong et al., 2024). These studies support the interface model’s distinction between sensory registration and cognitive access, but their direct relevance to postoperative recovery remains untested.

Electrophysiologically, propofol produces dose-dependent frontal alpha activity, slow oscillations, altered phase–amplitude relationships, and eventually burst suppression. Loss and return of behavioral responsiveness do not correspond to a single universal EEG threshold (Purdon et al., 2013). Measures of perturbational complexity and spontaneous signal diversity often fall during unconscious states, consistent with a reduced repertoire of differentiated responses (Casali et al., 2013; Sarasso et al., 2015).

### 5.2 Volatile anesthetics: preserved sensory networks amid higher-order disruption

Sevoflurane studies similarly show altered default-mode, ventral attention, frontal, and thalamic connectivity (Palanca et al., 2015). Simultaneous fMRI–EEG work found that moderate sevoflurane anesthesia reduced frontal and thalamic network integrity while primary sensory systems were comparatively preserved until deeper suppression (Ranft et al., 2016). Comparable dissociations between preserved early encoding and impaired higher-order processing have been reported in auditory paradigms under propofol (Liu et al., 2012) and in human intracranial recordings with the same drug (Krom et al., 2020). These findings align with a hierarchy in which late, recurrent, and integrative operations are more state-sensitive than initial encoding.

BOLD connectivity is sensitive to vascular physiology as well as neural activity (Golestani et al., 2016); this complicates interpreting cross-agent differences. Cross-agent comparisons therefore require end-tidal or plasma concentration, physiological recording, harmonized arousal endpoints, and ideally concurrent EEG.

### 5.3 Dexmedetomidine: sleep-like dynamics with important qualifications

Dexmedetomidine, an α2-adrenergic agonist, produces a state with some sleep-like behavioral and electrophysiological features, including arousability at lighter levels. Yet it is not equivalent to physiological sleep. Network-topological studies show substantial reconfiguration, and direct comparisons with propofol identify distinct EEG dynamics despite similar behavioral unresponsiveness (Akeju et al., 2014; Staquet et al., 2024). More recent work has further characterized the sleep-like but pharmacologically specific organization of dexmedetomidine sedation (Krause et al., 2026).

Clinically, dexmedetomidine has been associated with lower delirium incidence in selected trials and meta-analyses, particularly when used for postoperative sedation, but effects are context-dependent and accompanied by bradycardia and hypotension. A large randomized trial reported reduced POD after low-dose postoperative dexmedetomidine in older intensive-care patients (Su et al., 2016), whereas other perioperative regimens and comparator contexts have produced less consistent findings. Network preservation is a plausible mediator, but direct mediation evidence remains sparse; reduced opioid exposure, improved sleep, lighter sedation, and different hemodynamics are competing explanations.

### 5.4 Ketamine: dissociation is not simple disconnection

Ketamine illustrates why unconsciousness cannot be treated as a single network state. At anesthetic or subanesthetic concentrations, ketamine can produce dissociation, vivid internally generated experience, and altered sensory integration rather than uniformly reduced cortical complexity. Functional-connectivity studies show drug-specific changes in thalamocortical, frontoparietal, and sensory systems (Bonhomme et al., 2016), while cross-drug studies demonstrate that different anesthetics can disrupt frontal–parietal communication through distinct neurophysiological configurations (Lee et al., 2013). Thus, the interface model predicts agent-specific trajectories rather than a universal “connectivity suppression” pathway.

Clinical trials have not established ketamine as a reliable strategy for preventing delirium or postoperative cognitive decline. The PODCAST trial did not show that a single intraoperative ketamine dose prevented postoperative delirium and documented psychotomimetic adverse effects (Avidan et al., 2017). Conversely, a trial of subanesthetic S-ketamine during hip or knee arthroplasty under neuraxial anesthesia reported less POD (8.1% versus 20.4%) but more hallucinations, dizziness, and nightmares (Zhu et al., 2026). Potential benefit and psychotomimetic harm should therefore be interpreted in relation to formulation, regimen, and clinical context. In older patients, baseline cognition, frailty, and coadministered sedatives warrant attention; these trials do not establish an age-by-treatment interaction.

### 5.5 Depth, burst suppression, and neural susceptibility

Processed EEG indices, including BIS, summarize complex, age-dependent signals in a single number calculated by a proprietary algorithm. The raw EEG is needed to assess spectra, suppression, complexity, and connectivity independently (Purdon et al., 2013; Ranft et al., 2016; Shao et al., 2020). Mechanistic trials must therefore retain raw waveforms and document acquisition settings and artifacts so that analyses can be reproduced. Trials of EEG-guided anesthesia have yielded inconsistent delirium results. Earlier studies suggested that bispectral or depth-guided management could reduce anesthetic exposure and postoperative delirium or cognitive dysfunction (Chan et al., 2013; Radtke et al., 2013). ENGAGES found that EEG-guided anesthetic administration reduced exposure and burst suppression but did not significantly reduce delirium (Wildes et al., 2019). In contrast, the BALANCED delirium substudy found less POD with a BIS target of 50 than 35 (19% versus 28%; odds ratio 0.58, 95% CI 0.38–0.88) (Evered et al., 2021). Differences in protocols, achieved depth separation, patient risk, delirium ascertainment, cointerventions, and the biological meaning of the monitored index complicate comparison.

The more defensible interpretation is that EEG reveals neural susceptibility, not merely delivered dose. Excessive slow-wave activity or burst suppression at modest concentrations may identify a brain with reduced reserve; prolonged suppression may also be a modifiable exposure. Contemporary cohort studies link intraoperative spectral features, connectivity, and suppression patterns to POD, but predictive performance and causal direction vary (Fritz et al., 2016; Fritz et al., 2020; Shao et al., 2020; Guay et al., 2023; Neuner et al., 2023; Acker et al., 2024; Park et al., 2025; Xu et al., 2025; Fang et al., 2026; Van der A et al., 2026). Future trials should therefore test explicit EEG phenotypes and network mediators rather than assuming that reducing a proprietary index alone restores cognition.

### 5.6 From transient state change to postoperative recovery

Anesthesia-state studies suggest which processes may be affected, but the causes of persistent PND remain uncertain. The next question is whether brain networks recover normally after anesthesia. This requires direct measurement during recovery, because emergence may not retrace induction. We propose testing whether postoperative stressors disrupt network recovery even after wakefulness returns.

## 6 Surgery and the postoperative milieu: convergent perturbations of the interface

### 6.1 Neuroimmune signaling and blood–brain barrier function

Tissue injury activates innate immune pathways and releases cytokines, damage-associated molecular patterns, and acute-phase mediators. Experimental models show that interleukin-1β, tumor necrosis factor, and microglial responses can impair hippocampal plasticity and memory after surgery (Cibelli et al., 2010; Terrando et al., 2010; Feng et al., 2017). More recent mechanistic work describes coordinated peripheral and central immune responses rather than a simple passive “spillover” of inflammation into the brain (Yang et al., 2020). Human studies associate perioperative inflammatory markers with delirium or cognitive decline, but effect sizes are variable and temporality, comorbidity, infection, and surgical magnitude complicate causal interpretation (Subramaniyan and Terrando, 2019).

Blood–brain barrier and endothelial dysfunction offer a route by which systemic stress can alter neural homeostasis (Devinney et al., 2023). Yet neuroinflammation is not a complete phenotype-level explanation. Cytokines can alter neuromodulation, synaptic gain, glial metabolic support, and neurovascular coupling across many circuits. The interface model specifies a systems-level consequence: inflammatory signaling may reduce the precision and stability of long-range communication, amplify interoceptive salience, and impede the re-segregation of attention and association networks. This is plausible but requires simultaneous inflammatory and network measurements to establish mediation.

### 6.2 Cerebral perfusion, oxygen delivery, and neurovascular coupling

Hypotension, hypoxemia, anemia, hypocapnia or hypercapnia, embolic events, and impaired autoregulation can threaten a brain with limited vascular reserve. Observational studies associate intraoperative hypotension with adverse organ outcomes, but definitions and causal estimates vary (Hirsch et al., 2015). Individualized blood-pressure management has reduced organ dysfunction in some trials (Futier et al., 2017), yet evidence specifically linking such strategies to PND remains inconsistent (Bright et al., 2026). A universal pressure threshold ignores baseline hypertension, cerebrovascular disease, collateral capacity, anesthetic agent, and the duration and pattern of exposure.

Perfusion also affects measurement. BOLD connectivity is sensitive to carbon dioxide and cerebrovascular reactivity (Golestani et al., 2016). Arterial spin labeling provides perfusion information (Alsop et al., 2015). Combining it with physiological measurements may help assess how much of a BOLD change is attributable to vascular factors. New diffusion-restricted lesions or infarcts should be analyzed separately from functional dysconnectivity because direct tissue injury and state-dependent network failure may coexist but imply different interventions.

### 6.3 Pain, opioids, and interoceptive competition

Acute pain captures attention, disrupts sleep, enhances threat monitoring, and changes salience, default-mode, and control-network connectivity. Chronic-pain imaging demonstrates that individual pain experience depends on distributed networks rather than nociceptive pathways alone (Kucyi and Davis, 2015). In older adults, undertreated pain and high opioid exposure can both contribute to delirium; the clinically relevant target is adequate, opioid-sparing analgesia without excessive sedation. Population studies also associate pain with cognitive burden, but directionality is complex (Moriarty et al., 2011).

The interface account treats pain as high-precision internal evidence that competes with external orientation and goal-directed processing. Opioids provide analgesia but can also depress arousal (Brown et al., 2010). Regional analgesia, non-opioid adjuncts, and patient-specific titration may therefore support cognition through several routes. Direct imaging studies that demonstrate network mediation are still uncommon, so analgesic strategy should not be ranked solely on theoretical connectivity effects.

### 6.4 Sleep, circadian disruption, and environmental disorientation

Sleep disturbance is common before and after surgery and is associated with delirium and poorer cognitive recovery (Wang et al., 2021). Sleep loss changes slow-wave activity, synaptic homeostasis, attention, and default-mode/control-network interactions. Pain, ward interruptions, nocturnal light, medications, and circadian phase shifts compound one another. The relationship is bidirectional: prodromal delirium disrupts sleep, while disrupted sleep may increase vulnerability.

Environmental sensory conditions are mechanistically relevant but easily oversimplified. Removing spectacles or hearing aids reduces reliable input; alarms and frequent care produce overload; isolation and immobility reduce orienting cues; masks and unfamiliar voices impair speech and face integration. The goal is not maximal stimulation. It is a stable, interpretable, circadian-appropriate sensory environment with adequate vision and hearing, clear communication, mobility, sleep protection, and orientation. Multicomponent programs such as the Hospital Elder Life Program reduce delirium by addressing several vulnerabilities simultaneously (Inouye et al., 1999; Hshieh et al., 2015; Hshieh et al., 2018). Their efficacy supports systems-level prevention, although it cannot isolate a specific sensory–cognitive mechanism.

### 6.5 Neuronal injury markers and network dysfunction are complementary

Postoperative increases in neurofilament light, tau, and other neural-injury markers are reported in subsets of patients and may correlate with cognitive outcomes (Casey et al., 2020; Fong et al., 2020; Ballweg et al., 2021). These markers should not be treated as interchangeable with network dysfunction. A patient may show substantial but reversible functional reconfiguration without measurable axonal injury, while focal or diffuse injury may occur without a particular cognitive syndrome. Moreover, concentrations depend on baseline neurodegeneration, renal function, blood volume, timing, assay, and procedure.

The strongest test is longitudinal mediation with temporally ordered measurements, not cross-sectional correlation between one biomarker and one network metric.

## 7 Clinical phenotypes and time: what exactly should imaging explain?

### 7.1 Emergence phenomena and residual drug effect

Residual drug effects and physiological factors influence emergence and can complicate cognitive assessment (Brown et al., 2010). Imaging is rarely feasible in this interval, so assessment relies particularly on EEG, behavioral responsiveness, and the drugs administered. Emergence-related disturbances may involve some of the same network processes as POD. A diagnosis of delirium, however, requires the characteristic disturbances of attention and awareness and a fluctuating course, assessed with immediate recovery factors taken into account.

### 7.2 Postoperative delirium

POD develops over hours to days and fluctuates. Hyperactive, hypoactive, and mixed motor presentations can reflect different clinical manifestations of a common syndrome, but may also have partially distinct physiological contributors. Hypoactive delirium is particularly likely to be missed without repeated structured assessment (Wilson et al., 2020). Imaging biomarkers should be linked to temporally proximate delirium status and severity; a scan obtained after resolution addresses vulnerability or recovery, not necessarily the neural state of active delirium.

Delirium is a network-level disorder in contemporary models, involving arousal, attention, sensory interpretation, and integration of internal and external context (Wilson et al., 2020). Its association with later cognitive decline is robust at the population level, but three causal structures remain plausible and may coexist: delirium contributes to injury or accelerates decline; delirium is a marker of an insult that independently causes decline; and delirium reveals pre-existing pathology. Preoperative imaging and cognition are therefore indispensable.

### 7.3 Delayed neurocognitive recovery and postoperative neurocognitive disorder

Objective postoperative cognitive decline requires a baseline and an appropriate statistical definition. Single screening tools are insufficient to characterize domain-specific change, and repeated testing introduces practice effects. Reliable-change or normative approaches should include non-surgical controls when feasible, or at minimum account for age, education, test–retest effects, and baseline performance. The 30-day and 12-month nomenclature boundaries are clinically useful but do not imply abrupt mechanistic transitions (Evered et al., 2018).

Memory, attention, processing speed, and executive control may have different neural mediators. A global composite improves statistical power but can obscure meaningful dissociations. The interface model predicts, for example, that impaired early sensory fidelity and attention can secondarily reduce memory encoding, whereas persistent hippocampal or default-mode dysfunction may impair memory despite recovered sensory processing. Imaging should therefore be related to both domain composites and functional outcomes rather than to a single binary PND label.

### 7.4 Pre-existing impairment and unmasked neurodegeneration

Many older surgical patients have mild cognitive impairment, frailty, sleep disorders, depression, or subclinical neurodegeneration before surgery. Preoperative screening is not merely risk stratification; it defines the denominator for postoperative change. Alzheimer-type cortical atrophy has been linked to delirium risk in some imaging cohorts (Racine et al., 2017), and apolipoprotein E genotype may modify cognitive trajectories after surgery (Browndyke et al., 2021), but findings are not uniform. Biomarker-positive neurodegeneration could influence network reserve, inflammation, and recovery without making surgery the primary cause of long-term decline.

## 8 Direct human perioperative evidence: what each modality contributes

The perioperative imaging literature is expanding but remains fragmented. Studies differ in surgery, age, urgency, anesthetic exposure, delirium assessment, cognitive definition, imaging time, preprocessing, and statistical scale. Sample sizes are often modest, postoperative attrition is informative rather than random, and many findings lack external replication. Table 4 maps representative human MRI and EEG publications, explicitly noting that several reports reuse the same cohorts and therefore do not constitute independent replications. Several recurrent observations nevertheless support a vulnerability-plus-reconfiguration framework more strongly than a focal-lesion model.

**Table 4:** Representative human perioperative MRI and EEG studies.

Study (author–year citation; PMID)	Population/N	Timing	Modality/outcome	Principal observation	Interpretation	Major limitation
Cavallari et al. (2016) PMID 26920674	Elective surgery; age ≥70; N = 136; POD = 29	Preoperative	dMRI; POD incidence/severity	Distributed microstructural abnormalities including thalamus, basal forebrain, hippocampus, callosum, and association regions predicted POD	Preoperative diffusion abnormalities indicate structural vulnerability	DTI specificity; single cohort
Cavallari et al. (2017) PMID 28779010	SAGES subset; N = 113; POD = 25	Preoperative and 1 year	Longitudinal dMRI; cognition	POD occurrence/severity related to periventricular, frontal, and temporal diffusion change; diffusion change related to cognition	Delirium and long-term microstructural trajectory are linked	Cannot distinguish cause, correlate, or shared vulnerability
Hatano et al. (2013) PMID 24029014	Cardiac surgery; N = 130	Preoperative MRI; in-hospital POD	WMH	White-matter hyperintensity burden predicted delirium	Vascular structural burden may lower reserve	Retrospective chart review; cardiac-specific
Cavallari et al. (2015) PMID 25824618	Elective surgery; age ≥70; N = 146; POD = 32	Preoperative	T1/FLAIR; POD	WMH, whole-brain, and hippocampal volume were not significantly associated with POD incidence or severity	Global conventional burden is not sufficient	Dementia excluded; location/topology may matter
Racine et al. (2017) PMID 28846882	Elective surgery; age ≥70; N = 145	Preoperative	Cortical thickness; POD	Alzheimer-signature atrophy associated more clearly with delirium severity than incidence	Latent neurodegeneration may influence expression	Biomarker proxy; observational
Kyeong et al. (2018) PMID 29765105	Femoral-neck fracture; N = 57; POD = 25	Preoperative	T1, dMRI, rs-fMRI; POD	Multimodal structural and spontaneous-activity features contributed to an individual probability model	Vulnerability exists before surgery	Small, urgent-surgery cohort; model needs validation
Oh et al. (2019) PMID 31094211	Femoral-neck fracture; N = 58; POD = 25; active-POD scans = 14	Preoperative and active delirium / day 5–6 controls	rs-fMRI; POD state	Abnormal DMN–task-positive reciprocity and subcortical–striatal–cortical connectivity during POD	Active delirium is a distributed network state	Few active-POD scans; motion/medication
Tanabe et al. (2020) PMID 32499013	Surgical cohort; N = 70	Pre-, intra-/postoperative	MRI, EEG, inflammation; POD	POD occurred when slow activity spread posteriorly with connectivity breakdown; changes related to severity/inflammation	Slow-wave topography plus disintegration matters more than frontal slowing alone	Modest sample; complex multimodal analysis
Winterer et al. (2021) PMID 33846284	BioCog; preop N = 283, POD = 46; follow-up N = 178, prior POD = 19	Preoperative and 3–5 months	rs-fMRI; POD	POD group showed preoperative hyperconnectivity in orbitofrontal, accumbens, and hippocampal networks, followed by selected decreases	Hyperconnectivity may be compensation or dedifferentiation	Attrition; seed-limited analysis
Browndyke et al. (2017) PMID 27858963	Cardiac surgery N = 12; matched controls N = 12	Preoperative and 6 weeks	rs-fMRI and cognition	Preliminary postoperative connectivity changes covaried with cognition	Within-person change is measurable	Very small sample
Browndyke et al. (2018) PMID 29164774	Cardiac surgery N = 25; controls N = 26	Preoperative and 6 weeks	N-back fMRI; cognition	Posterior-cingulate degree centrality/local coherence changed with working-memory load	Task probes add process specificity	Performance and activation are coupled
Jiang et al. (2020) PMID 32617099	Non-cardiac surgery; N = 74; DNR = 16	Preoperative; cognition at 7–14 days	T1/rs-fMRI; DNR	Higher middle-cingulate ALFF and lower middle-cingulate–calcarine FC associated with DNR	Sensory–association interaction may index vulnerability	Nested case–control; no external validation
Jiang et al. (2021) PMID 34867266	Non-cardiac surgery; N = 74	Preoperative; early DNR	rs-fMRI machine learning	DMN, salience, executive, and limbic FC yielded high internal prediction performance	Distributed networks contain risk information	Small test set; likely optimistic performance
Jiang et al. (2022) PMID 36688167	Non-cardiac surgery; N = 74	Preoperative; early DNR	DMN whole-brain FC; machine learning	Reduced DMN connectivity with visual-processing regions characterized DNR	Directly motivates a sensory–cognitive interaction	Cohort overlap/reuse; needs external validation
Browndyke et al. (2021) PMID 34535274	Non-cardiac surgery; N = 140; imaging N = 52	Preoperative and 6 weeks	Cognition, CSF, rs-fMRI; APOE4	No uniform APOE4-related cognitive or connectivity trajectory	Genetic vulnerability effects may be modest or context-dependent	Secondary analysis; imaging subset
Gjini et al. (2024) PMID 37695013	N = 131 with ≥1 EEG; postoperative N = 110 (POD = 19); paired N = 102	Preoperative and POD period	High-density EEG oddball / DCM	Smaller mismatch negativity and DCM-inferred superior-temporal → auditory feedback; exploratory inflammation/severity links	Task-based evidence consistent with impaired predictive feedback	Hearing, opioid/sedative effects; model-dependent DCM; independent replication needed
Zhang G. et al. (2024) PMID 39298114	Frontal glioma surgery; N = 36 recruited	Pre- and postoperative	Gray-matter volume and resting-state FC; POD	Visual-network structural and functional alterations related to POD	Visual-network correlates; SC–FC coupling was not tested	Specialized neurosurgical population
Browndyke et al. (2024) PMID 38155462	Surgical aortic valve replacement; imaging N = 298; POD analysis N = 277; POD = 65	Early postoperative DWI	Infarct-related structural disconnection	POD associated with greater strategic disconnection from covert infarcts	Lesion topology matters more than “silent” label	Secondary trial analysis; cardiac-specific
Wang et al. (2025) PMID 40979560	Heart-valve surgery; N = 25 plus controls	Baseline, day 7, day 30	rs-fMRI, cytokines, cognition	Global left-frontal connectivity moderated inflammation–cognition association	Network reserve may modify biological insult	Small, hypothesis-generating cohort
Taylor et al. (2026) PMID 41475933	Major elective non-intracranial surgery; N = 120; POD = 31	Preoperative	400-region rs-fMRI; POD	Lower within-network salience/VAN, control, and DMN connectivity; visual and salience/VAN features classified POD with 68% accuracy	Vulnerability spans sensory, attention, and association levels	Internal accuracy; external validation needed; IPOD-B3 also contributes to Gjini et al.
Lammers-Lietz et al. (2026) PMID 41699935	Major surgery; N = 214; 3-month cognitive decline = 26 (source-defined POCD)	Preoperative and 3 months	VTA/SNc rs-fMRI; reliable-change cognition	Dopaminergic subnetwork connectivity components associated with later cognitive dysfunction	Neuromodulatory systems complement cortical networks	Exploratory components; nomenclature legacy
Ditzel et al. (2023) PMID 36261307	Strict-QC longitudinal sample N = 246; POD = 38	Preoperative and 3 months	rs-fMRI; TMT-B	Connectivity increased overall but decreased after POD; decrease associated with TMT-B decline	Recovery trajectory carries cognitive information	More than half of enrolled sample excluded after QC/missingness

The table intentionally includes positive, null, and apparently opposing findings. Rows represent publications, not independent cohorts; several analyses reuse the same parent cohort. Table 4 gives the matching author–year citation and PubMed ID for every publication. Rows identify publications rather than necessarily independent cohorts, because several analyses reuse parent cohorts. Entries summarize reported associations and do not imply causal effects. POCD is retained only as the source study’s legacy term for test-defined cognitive decline; it is not retrospectively equated with postoperative NCD under the 2018 nomenclature.

### 8.1 Structural MRI: burden is neither necessary nor sufficient

Conventional structural MRI can quantify global and regional atrophy, hippocampal volume, cortical thickness, white-matter hyperintensity burden, and overt lesions. Findings are mixed. In 146 dementia-free adults aged at least 70 years undergoing elective surgery, presurgical white-matter hyperintensity, total brain, and hippocampal volumes were not significantly associated with delirium incidence or severity (Cavallari et al., 2015). By contrast, a cardiac-surgery cohort linked white-matter hyperintensities to postoperative delirium (Hatano et al., 2013), and an Alzheimer-related cortical-thickness signature was associated with delirium severity, although not unequivocally with incidence, in another older elective-surgery cohort (Racine et al., 2017).

These results are not simply contradictory. Global lesion volume may be insensitive to strategically located or network-disconnecting pathology; associations may differ between cardiac and non-cardiac procedures; and excluding dementia narrows the range of structural burden. Structural MRI is likely most informative when topology, location, and connectivity consequences are considered. In patients undergoing surgical aortic valve replacement, infarct-related structural disconnection—not merely lesion volume—was greater among those with delirium, implicating frontal and temporal pathways despite clinically “silent” infarcts (Browndyke et al., 2024).

Structural measures are therefore best interpreted as reserve and injury context. A normal-appearing T1-weighted scan does not rule out microstructural, physiological, or functional vulnerability, while extensive atrophy or white-matter disease does not determine outcome (Cavallari et al., 2015, 2016).

### 8.2 Diffusion MRI: pre-existing dysconnectivity and longitudinal change

Diffusion MRI provides some of the strongest evidence that vulnerability precedes surgery. In the Successful Aging after Elective Surgery cohort, presurgical diffusion abnormalities in 136 dementia-free older adults were associated with delirium incidence and severity. Associations involved interhemispheric, cingulo-limbic, thalamic, basal-forebrain, cerebellar, and association pathways; several remained after adjustment for baseline cognition (Cavallari et al., 2016). This distributed pattern is consistent with reduced reserve across arousal, relay, memory, and integration systems rather than one critical tract.

Longitudinal data from 113 participants in the same program showed that delirium occurrence and severity were associated with one-year diffusion changes in periventricular, frontal, and temporal white matter, which also related to cognitive change (Cavallari et al., 2017). The design cannot determine whether delirium itself drove microstructural change, marked a shared insult, or identified brains already on a neurodegenerative trajectory. Nevertheless, it demonstrates why preoperative-only or postoperative-only imaging is inadequate for causal claims.

Diffusion measures have important limitations. Fractional anisotropy is sensitive but nonspecific; crossing fibers, extracellular water, edema, and scanner differences complicate interpretation. Tractography produces plausible streamlines, not direct axonal counts, and inter-algorithm variability can be substantial (Maier-Hein et al., 2017). Perioperative studies should include robust distortion and motion correction, model free water where justified, quantify new diffusion lesions separately, and report tractography choices transparently. Network analyses should test prespecified pathways and whole-connectome topology while controlling multiplicity.

### 8.3 Resting-state fMRI before surgery: vulnerability is distributed

Several preoperative resting-state studies identify functional phenotypes associated with subsequent POD. In 57 patients with femoral-neck fracture, multivariate structural and resting-state features differentiated those who later developed delirium, suggesting that vulnerability was present before the operation (Kyeong et al., 2018). In 120 adults older than 65 years undergoing major elective non-intracranial surgery, 31 developed POD. They showed lower preoperative within-network connectivity in salience–ventral attention, cognitive-control, and default-mode systems; visual and salience–ventral attention features contributed to a support-vector classifier with 68% reported accuracy (Taylor et al., 2026). This result is mechanistically informative but insufficient for individual clinical prediction without external validation. The coexistence of visual and higher-order network differences is directly relevant to a sensory–cognitive hierarchy.

Other cohorts show preoperative hyperconnectivity. In the BioCog study, 283 older patients underwent preoperative fMRI and 46 developed POD. Orbitofrontal, nucleus accumbens, and hippocampal networks were hyperconnected before surgery in patients who later developed delirium; among those returning for imaging 3–5 months later, selected connectivities decreased (Winterer et al., 2021). Hyperconnectivity may represent compensation for latent dysfunction, loss of network specificity, dopaminergic dysregulation, or a selection effect. The observation cautions against a universal “low connectivity equals risk” rule.

Resting-state prediction of delayed neurocognitive recovery has likewise highlighted interactions between association and visual systems. In a 74-patient cohort, delayed recovery at 7–14 days was associated with higher preoperative middle-cingulate activity and lower connectivity between middle cingulate and calcarine cortex (Jiang et al., 2020). Analyses of the same-sized cohort reported lower default-mode connectivity with visual-processing regions and high internal machine-learning performance (Jiang et al., 2021; Jiang et al., 2022). These models require independent, multisite validation: small test sets, feature selection, imbalance, and repeated use of a single cohort can inflate apparent accuracy. Their mechanistic value is stronger than their current readiness for individual clinical prediction.

### 8.4 Resting-state fMRI during delirium and recovery: network balance changes over time

Imaging during active delirium is difficult and therefore rare. In patients with femoral-neck fracture, serial resting-state fMRI revealed altered default-mode interactions, reduced normal opposition between default-mode and task-positive regions, and abnormal subcortical–striatal–cortical connectivity during delirium (Oh et al., 2019). The small number scanned during active episodes limits generalization, and motion or medication effects remain concerns, but the findings support disrupted reciprocity among internally oriented, control, salience, and subcortical systems.

Recovery studies provide a longer temporal view. In a multicenter cohort, stringent motion control left 246 participants with preoperative and three-month fMRI; 38 had developed POD. Connectivity strength increased after surgery in the overall cohort but decreased after POD, and decreasing connectivity was associated with worsening Trail Making Test performance (Ditzel et al., 2023). In cardiac surgery, small longitudinal studies have reported changes in resting connectivity and task-related posterior-cingulate centrality at approximately six weeks (Browndyke et al., 2017; Browndyke et al., 2018). APOE4-related differences were not uniformly detectable in cognition or network trajectories, underlining the need for adequately powered interaction analyses (Browndyke et al., 2021).

A 2025 longitudinal cardiac-surgery study further suggested that global left-frontal connectivity moderated the relationship between postoperative inflammation and cognitive performance (Wang et al., 2025). Its small sample makes the result hypothesis-generating, but the design exemplifies a more mechanistic question: not merely whether inflammation and cognition correlate, but whether network reserve changes their relationship. A 2026 BioCog analysis of 214 patients implicated pre- and postoperative connectivity of dopaminergic midbrain subnetworks in three-month cognitive dysfunction, extending the framework beyond canonical cortical networks (Lammers-Lietz et al., 2026).

### 8.5 Task fMRI and sensory probes: process specificity remains underused

Resting-state fMRI is feasible and broad, but it cannot identify whether an abnormal edge reflects sensory encoding, salience detection, working-memory maintenance, or unrelated spontaneous state. Task paradigms can provide process specificity. N-back studies after cardiac surgery have identified altered posterior-cingulate centrality and local coherence as memory load increases (Browndyke et al., 2018). However, complex tasks selectively exclude frail or cognitively impaired patients and conflate performance with neural activation.

Short, adaptive sensory tasks may be more suitable. Auditory oddball, speech-in-noise, audiovisual congruence, somatosensory prediction-error, or attention-reorienting paradigms can separate early encoding from later mismatch, feedback, and access. A longitudinal study in patients with frontal glioma linked gray-matter volume and resting-state visual-network connectivity to POD (Zhang G. et al., 2024). These measurements did not constitute diffusion-based SC–FC coupling. The specialized neurosurgical population and potential tumor-related reorganization limit generalization, but the study demonstrates that sensory networks can be interrogated directly rather than treated as passive comparators.

### 8.6 EEG: temporal resolution, bedside feasibility, and mechanistic leverage

EEG measures neural dynamics at the timescale of information flow and can be repeated when MRI is unsafe or impractical. Increased delta or slow-wave activity and reduced functional connectivity are common during delirium, but frontal slowing also occurs after surgery in patients without delirium. In a multimodal cohort of 70 patients, delirium emerged when slow-wave activity extended from frontal to posterior regions alongside connectivity breakdown; connectivity changes related to inflammation and delirium severity (Tanabe et al., 2020). Preoperative alpha features also differed, suggesting baseline susceptibility.

Evoked responses can test the sensory–cognitive interface more directly. Of 131 patients with EEG data, 110 contributed postoperative data (19 with POD), and 102 had paired recordings. POD was associated with smaller mismatch negativity and reduced model-estimated feedback from left superior temporal to primary auditory cortex; feedback strength related inversely to delirium severity, with exploratory, uncorrected associations with inflammation (Gjini et al., 2024). This supports impaired top-down sensory prediction in delirium, although model-derived connectivity does not demonstrate a causal mechanism. Replication, medication adjustment, and clarification of whether changes precede or follow delirium onset are still needed. Analyses should record hearing thresholds, stimulus audibility, and hearing-aid use, and account for opioid dose and timing, other sedatives, pain, and arousal when interpreting postoperative auditory responses.

Intraoperative EEG studies examine spectral power, connectivity, complexity, suppression, and age-adjusted signatures. These measures have been associated with delirium risk or suppression susceptibility, although they are not interchangeable biomarkers (Fritz et al., 2016; Shao et al., 2020; Xu et al., 2025). More consistent reporting would help comparison across studies. Proprietary indices should be accompanied by raw waveforms, with spectra provided as complementary derived data. Reports should describe electrode density, montage, and artifact handling, and include preoperative or postoperative awake baselines where possible.

### 8.7 Perfusion, fNIRS, and multimodal monitoring

Arterial spin labeling can quantify cerebral perfusion without contrast and may help interpret BOLD changes, but signal-to-noise and transit-time effects complicate quantification (Alsop et al., 2015). fNIRS offers portable cortical oxygenation and hemodynamic measurement, although limited depth and systemic contamination constrain inference (Yücel et al., 2021). These methods are best used to test explicit physiological mediators rather than as unvalidated substitutes for neural connectivity.

Structural MRI and diffusion characterize reserve and injury; BOLD and perfusion describe spatial organization and vascular context; EEG tests temporal routing and arousal; blood biomarkers index inflammation or neural injury; and cognition defines behavioral expression.

## 9 Multimodal integration: SC–FC coupling as a candidate measurement

### 9.1 A bridge between relatively stable structure and rapidly changing function

SC–FC coupling measures how closely functional coordination corresponds to anatomical connections. It may help relate baseline reserve to changes in network organization across perioperative states, although its value in this setting remains unvalidated. The sensory–cognitive account can also be tested using EEG, sensory tasks, and network measures without an SC–FC coupling analysis.

Regional SC–FC coupling is often estimated by correlating the structural weights incident on a region with the corresponding functional-connectivity edges, after prespecifying how absent edges, distance, weight transformation, and sign will be handled (Gu et al., 2021; Fotiadis et al., 2024). Edge-wise, network-level, graph-signal, and subject-level approaches address different questions and cannot be used interchangeably. Perioperative studies also need to distinguish between-person differences at a single time point from within-person changes in coupling. Only the latter directly describe reconfiguration, and even these changes may reflect altered hemodynamics unless physiological or electrophysiological findings support a neural interpretation. Vascular effects on BOLD connectivity are therefore relevant to the interpretation of SC–FC coupling (Golestani et al., 2016). ASL can provide perfusion measurements (Alsop et al., 2015), but cannot establish neural causation on its own; we propose physiological recording and EEG as complementary checks. Figure 2 outlines the analysis from paired structural and functional connectomes to a spatial coupling profile and longitudinal clinical testing. Table 5 summarizes the information provided by each complementary modality and its limitations.

**Figure 2:**
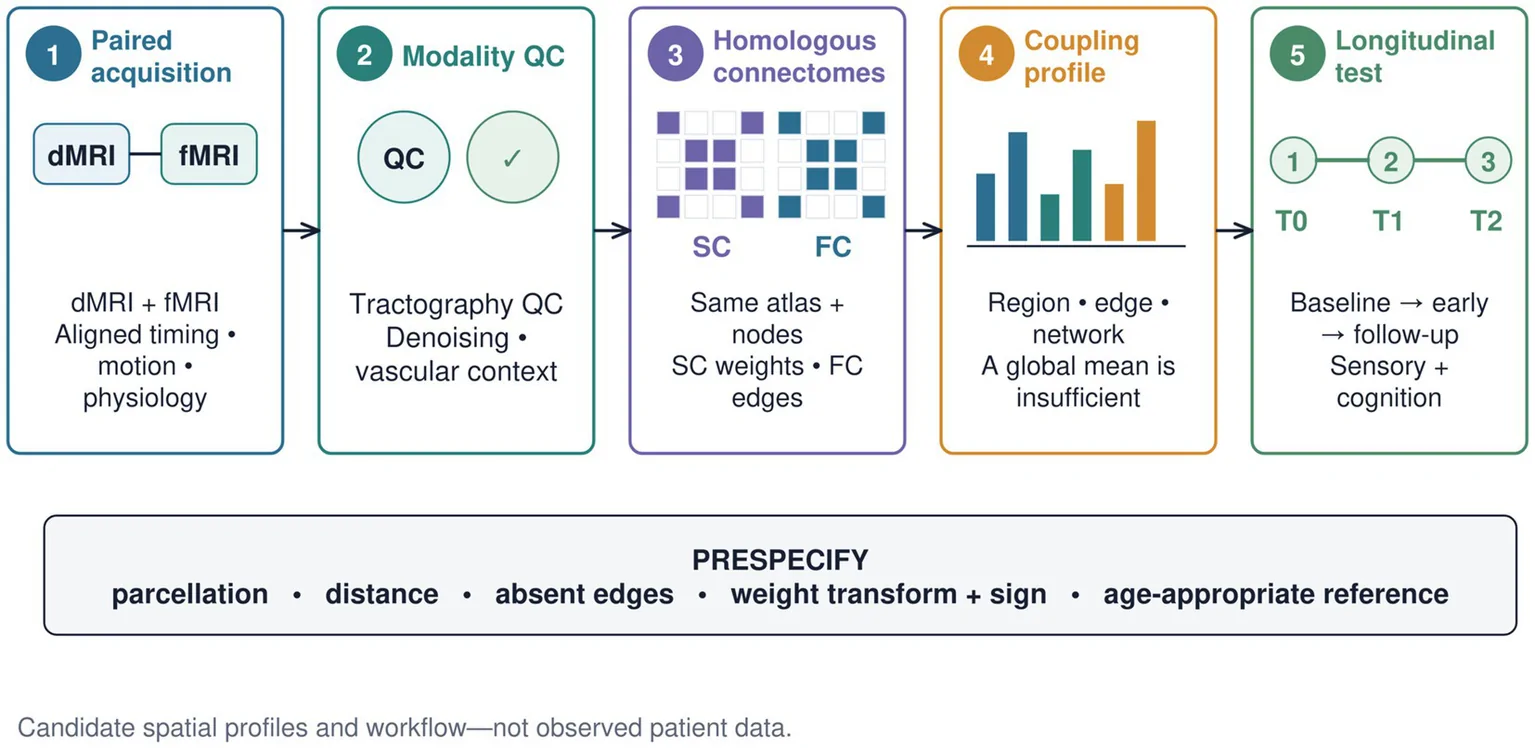
Analytical workflow for spatially resolved structural–functional connectivity coupling. Paired diffusion MRI and functional data are converted into structurally and functionally homologous connectomes after modality-specific quality control. Regional, edge-wise, or network-level coupling is then estimated with prespecified handling of distance, absent edges, weight transformation, sign, parcellation, and age-appropriate reference. Longitudinal change is related to sensory and cognitive phenotypes while testing vascular and electrophysiological alternatives. The schematic represents a workflow and candidate spatial profiles, not observed patient data. SC–FC coupling is an optional research measure; its incremental value for POD remains unvalidated.

**Table 5:** Multimodal measures, inferential value, and principal confounds.

Measure	Primary question	Representative metrics	Resolution / setting	Strength	Principal confounds and safeguards
Sensory phenotyping	How reliable is afferent evidence?	Acuity, contrast, thresholds, speech-in-noise, aid use	Bedside / clinic	Directly tests effect modification	Aid availability, language, fatigue; standardize conditions
Cognitive phenotyping	Which domain and functional outcome changed?	Attention, speed, executive, working and episodic memory, function	Bedside / laboratory	Defines behavioral expression	Practice, education, mood, pain; alternate forms and reliable change
T1-weighted MRI	What reserve/atrophy exists?	Thickness, volume, subcortical segmentation	Millimeters; pre/follow-up	Stable anatomical context	Scanner/site, segmentation, hydration; visual QC
FLAIR/susceptibility	What vascular burden exists?	WMH, lacunes, microbleeds	Millimeters	Clinically interpretable burden	Rating versus volume, lesion topology; harmonized segmentation
DWI/diffusion MRI	Is there acute injury or latent dysconnectivity?	Acute lesions; FA/MD/free water; tractography; network efficiency	Millimeters; minutes	Structural scaffold and strategic disconnection	Motion, crossing fibers, algorithm variability; report full pipeline
Resting-state fMRI	How are large-scale systems coordinated?	Within/between FC, segregation, gradients, dynamics	Seconds–minutes; MRI	Broad network coverage without task	Motion, physiology, arousal, vascular coupling; censor and record
Task fMRI	Which operation is abnormal?	Sensory evoked response, oddball, congruence, N-back	Seconds; MRI	Process specificity	Performance and selection bias; use short adaptive tasks
ASL/perfusion	How does perfusion relate to the BOLD change?	CBF, transit time	Minutes; MRI	Quantitative hemodynamic context	Low SNR, transit time, vascular disease, CO_2_; align physiology/EEG; ASL alone cannot establish neural origin
EEG	How do arousal and information routing change?	Spectra, suppression, connectivity, complexity, evoked feedback	Milliseconds; bedside/intraoperative	Temporal resolution and feasibility	Artifact, montage, proprietary indices; preserve raw data
fNIRS / cerebral oximetry	Can cortical hemodynamics be monitored portably?	HbO/HbR, regional oxygenation	Seconds; bedside/OR	Repeatable when MRI infeasible	Superficial/systemic contamination, limited depth; short-channel correction
Blood biomarkers	What biological perturbation or injury occurred?	Cytokines, NfL, tau, glial markers	Hours–days	Scalable molecular context	Timing, renal function, dilution, nonspecificity; serial aligned sampling
SC–FC coupling	How is function expressed on structural scaffold?	Regional, edge-wise, network coupling; structural decoupling	Cross-modal	Candidate measure linking structural and functional organization	Parcellation, tractography, distance, metric choice; prespecify direction

Directions are network-, region-, method-, and time-specific.

### 9.2 The relevant unit is a spatial profile, not one global number

Because structure–function relationships vary along the cortical hierarchy, whole-brain average coupling can conceal opposite regional effects (Gu et al., 2021; Valk et al., 2022). A rigorous perioperative analysis should prespecify at least three scales:

- Unimodal coupling, where relatively tight alignment may support reliable sensory transmission;
- Intermediate network coupling, including salience, attention, and control systems that coordinate flexible routing;
- Transmodal coupling or decoupling, where an appropriate degree of functional freedom may support contextual integration.

Time-resolved coupling approaches may capture transient configurations, but their reliability and sensitivity to windowing require careful validation (Liu et al., 2022). The expected direction must be stated for each region and time point. “Normalization” should mean movement toward a well-defined, age-appropriate reference profile associated with better function—not simply an increase.

### 9.3 Integration with gradients and dynamics

Gradient analysis reduces complex connectivity into continuous axes of organization (Margulies et al., 2016) and is well suited to testing whether perioperative perturbation compresses the separation between sensory and transmodal systems.

Dynamic functional connectivity offers complementary information about the repertoire and dwell time of network states. Yet dynamic analyses can create apparently meaningful states from sampling noise, motion, or arousal variation; strong null models and test–retest reliability are essential (Hindriks et al., 2016).

### 9.4 What multimodal convergence would—and would not—establish

Convergent findings across diffusion MRI, fMRI, and EEG strengthen a network interpretation when they are anatomically and temporally aligned.

Even when findings from different modalities agree, causal interpretation requires within-person changes measured in temporal order, appropriate controls, and explicit assumptions about confounding. An intervention that changes the proposed network mediator would provide stronger support.

## 10 An integrated two-hit, precision-failure, and failed-recovery model

Drawing on established accounts of vulnerability, perturbation, and recovery, we describe four proposed stages and three possible recovery trajectories (Figure 3). The stages remain hypothetical; they have not been observed as a sequence in POD.

**Figure 3:**
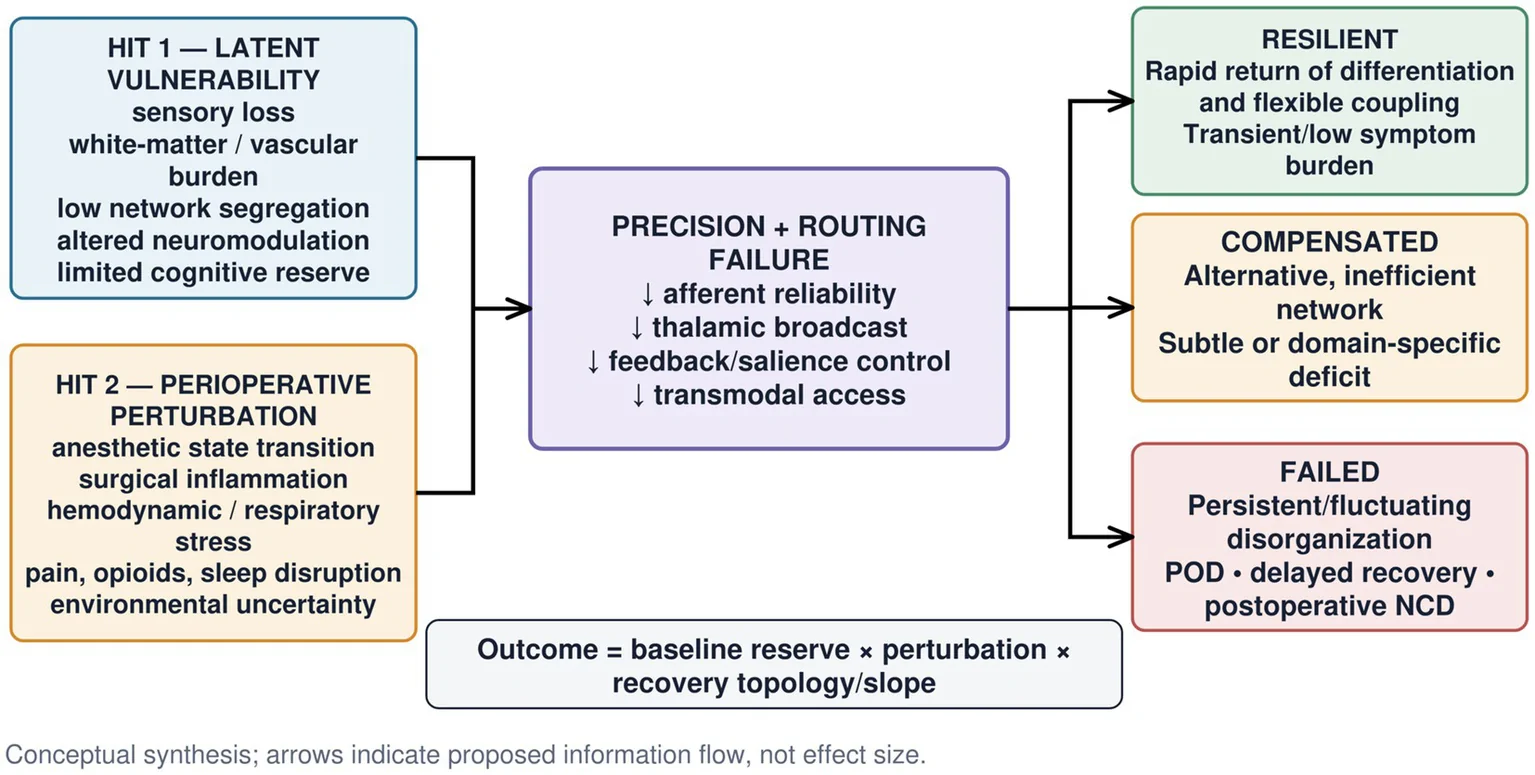
Two-hit, precision-failure, and failed-recovery model. Baseline sensory, structural, vascular, and cognitive vulnerability may interact with anesthesia, surgical inflammation, physiological stress, pain, sleep disruption, medication, and environmental uncertainty. These influences can impair afferent precision, thalamic broadcasting, feedback, salience control, and transmodal access. Subsequent reconfiguration is resilient, compensated, or failed and may be associated with phenotype-specific outcomes across different time windows. The multiplicative schematic illustrates the model and has not been fitted as a causal equation. POD, delayed recovery, and later NCD are distinct possible outcomes and need not occur in sequence.

### 10.1 Stage 1: latent vulnerability

The model proposes that established vulnerabilities—including aging, sensory impairment, vascular disease, neurodegeneration, and frailty (Wilson et al., 2020)—reduce the fidelity and flexibility of the sensory–cognitive hierarchy. These features need not cause daily-life impairment because stable environments and compensatory strategies support function.

### 10.2 Stage 2: acute perioperative disconnection

Anesthetic drugs reduce arousal and reconfigure thalamocortical, feedback, and transmodal interactions (Lee et al., 2013; Malekmohammadi et al., 2019). Surgery adds inflammation, hemodynamic stress, pain, embolic or direct injury risk, and metabolic disturbance. Sensory aids may be removed and environmental uncertainty increases. We hypothesize that these disturbances reduce the reliability of bottom-up sensory information and disrupt precision weighting. They may also increase the salience of internal bodily signals and restrict access to distributed cognitive processing.

### 10.3 Stage 3: re-emergence and network reconfiguration

Eye opening or command following indicates behavioral emergence, but brain networks may still differ from their preoperative state. Inflammation, sleep loss, residual drug effects, pain, and ambiguous sensory input continue to affect this period. Network recovery therefore needs to be considered beyond the first signs of awakening.

### 10.4 Stage 4: phenotype expression

Unstable or regionally abnormal reconfiguration may contribute to the fluctuating disturbances of attention and contextual integration seen in POD. Persistent disruption, direct injury, or unmasked neurodegeneration may contribute to later cognitive disorders, which need not follow the same course as POD. The clinical presentation depends on the processes affected as well as the overall magnitude of network change.

### 10.5 Three recovery trajectories

Resilient recovery is characterized by transient, exposure-appropriate disconnection followed by rapid restoration of network differentiation, feedback, and domain-specific cognition. Compensated recovery shows abnormal baseline or early postoperative connectivity, possibly including hyperconnectivity, but recruits alternative pathways sufficiently to restore behavior; it may consume reserve and predict vulnerability to later stress. Failed or maladaptive recovery shows persistent reduction of state repertoire, inappropriate coupling along the hierarchy, loss of feedback, or disintegration across sensory, salience, and control systems, accompanied by delirium or cognitive decline. Figure 4 depicts these as explicitly conceptual trajectories rather than observed patient-level curves.

**Figure 4:**
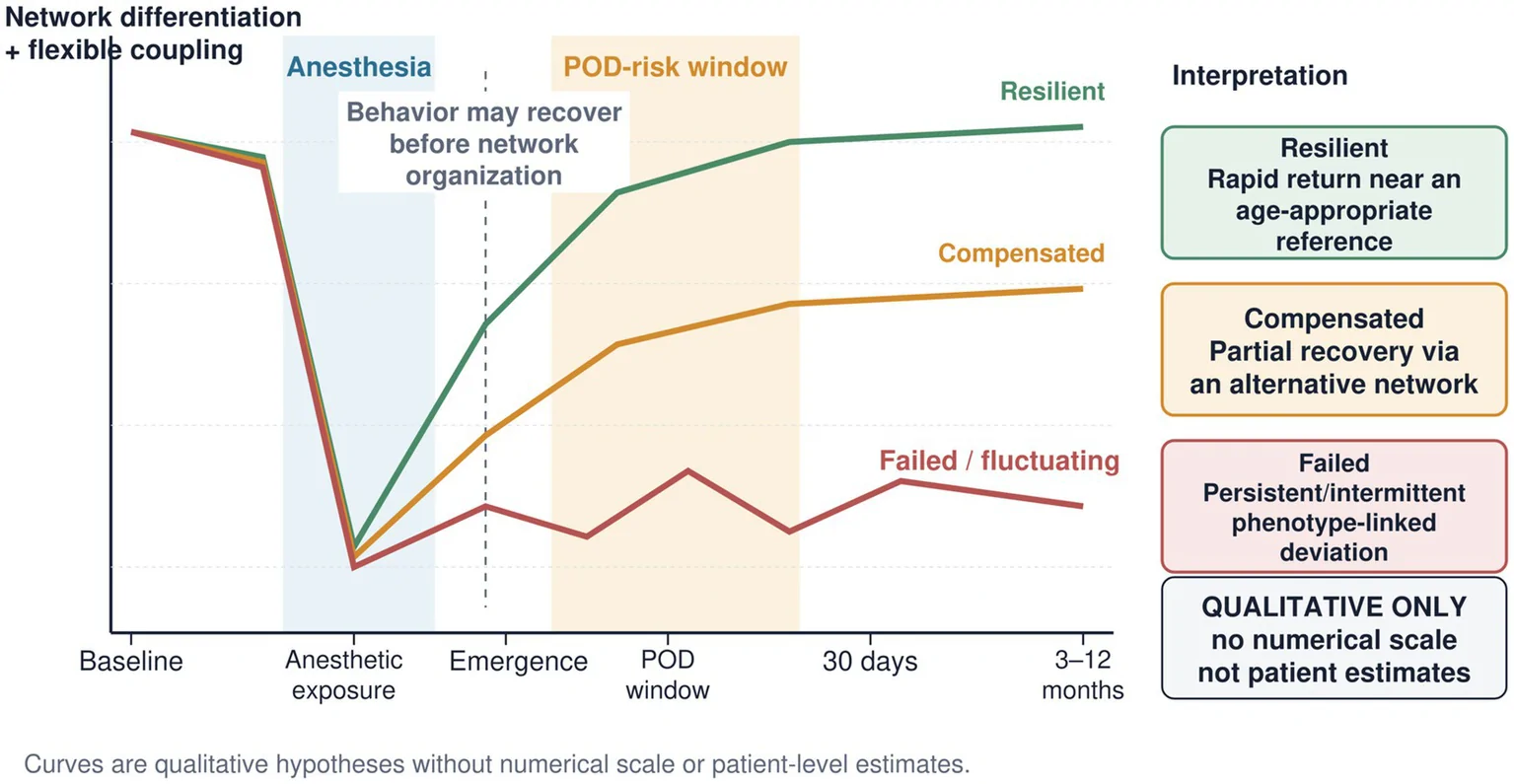
Conceptual perioperative recovery trajectories. Behavioral emergence is distinguished from restoration of cognitive network organization. Resilient recovery rapidly restores differentiation and flexible coupling; compensated recovery relies on alternative or inefficient configurations; failed recovery remains persistently or intermittently abnormal. Curves are qualitative hypotheses without numerical scale and should not be interpreted as patient-level estimates. These proposed patterns have not been established as patient classes, and POD does not inevitably lead to later NCD.

### 10.6 Falsifiable predictions

Table 6 gives a study design for each prediction and specifies findings that would argue against it. These tests need reliable measurements, sufficient variation in exposure, and adequate statistical power. A negative result for one candidate measure would challenge that measure without necessarily invalidating the wider framework:

1 Interaction prediction: baseline sensory function and network reserve should modify the association between perioperative exposure and cognitive outcome. Failure to detect the prespecified interaction in a sufficiently powered study would weaken this prediction. Additive effects would still be compatible with broader vulnerability models.
2 Hierarchy prediction: transmodal and feedback interactions should show greater state sensitivity, while persistent symptoms during POD should involve impaired reintegration across sensory, salience–attention, and association levels rather than isolated global suppression.
3 Trajectory prediction: within-person recovery slopes should explain delirium burden and domain-specific cognitive change better than a single preoperative or postoperative scan.
4 Optional coupling prediction: spatially resolved SC–FC deviation from an age-appropriate hierarchy should predict recovery more accurately than global structural or functional metrics alone.
5 Phenotype prediction: attention and perceptual instability should relate more strongly to thalamic, salience, and feedback measures; memory decline should additionally depend on hippocampal–default-mode and encoding-related changes.
6 Mediation prediction: an intervention that reduces POD or cognitive decline through the proposed mechanism should measurably alter the predicted network trajectory, and mediation should remain after exposure and baseline vulnerability are modeled.
7 Specificity prediction: network changes attributable only to residual drug level, motion, or vascular BOLD effects should not persist after those factors are addressed or reproduce in EEG and behavior.

**Table 6:** Falsifiable predictions and study designs for the proposed model.

Prediction	Primary design	Key variables	Supportive result	Result that weakens the claim
Reserve × exposure interaction	Prospective cohort or randomized exposure contrast	Sensory function, baseline networks, quantified dose/physiology, cognition	Baseline reserve modifies exposure–outcome association	Precise estimates exclude a meaningful interaction when measurement is reliable and exposure contrast is adequate
Hierarchical sensitivity	Within-person anesthetic and postoperative sampling	Unimodal, intermediate, transmodal, and thalamic metrics	Feedback/transmodal measures change earlier or recover later than encoding	Uniform global suppression explains all levels equally
Recovery trajectory	≥3 longitudinal time points	Within-person network slope, POD burden, cognitive domains	Trajectory outperforms single scans and relates to phenotype	Single baseline value fully explains outcome; slope adds no value
Spatial SC–FC profile	Paired dMRI–fMRI with age-appropriate reference	Regional coupling along principal gradient	Hierarchical deviation and recovery add out-of-sample value	Global or regional coupling adds nothing beyond clinical/MRI baseline
Phenotype specificity	Domain-based cognition plus sensory tasks	Attention/perception, memory, thalamic/salience, and hippocampal/DMN metrics	Distinct process–network associations reproduce	All domains show nonspecific association with one global metric
Intervention mediation	Randomized mechanistic trial	Treatment, target network change, cognition/POD	Intervention changes predicted mediator and indirect effect is present	Clinical benefit occurs without target engagement or mediator relation
Cross-modal convergence	Synchronous/temporally aligned MRI, EEG, physiology	Anatomical, hemodynamic, and electrical measures	Anatomically aligned changes persist after motion/physiology control	Effect disappears after confound control or contradicts direct electrophysiology
Sensory modifier	Stratified cohort or sensory-bundle trial	Hearing/vision, aids/environment, network, and cognition	Sensory status modifies network–outcome relation or bundle engages target	Sensory measurement and intervention show no modifier or target effect

A useful model must specify what result would weaken it, not only what result would appear supportive. A negative result can weaken a prediction only if the study has reliable measurements, appropriate timing, sufficient exposure contrast, and adequate statistical precision. Statistical nonsignificance alone is insufficient.

Failure of SC–FC coupling to add out-of-sample value beyond age, cognition, frailty, sensory status, and conventional MRI would refute its proposed biomarker utility even if the broader interface concept remains viable. Likewise, if sensory measures do not modify network–outcome relationships, the model should be narrowed to a more general hierarchy-of-integration account.

## 11 Clinical implications: actionable now, plausible, and not yet ready

The framework should not be mistaken for a recommendation to obtain clinical fMRI in routine perioperative care. The studies reviewed here have not established a network metric with adequate validation and clinical utility for diagnosing or predicting PND in individual patients. Its immediate value is to organize proven and low-risk clinical practices, identify potentially modifiable exposures, and improve the design of mechanistic trials.

### 11.1 Actions supported by current clinical evidence

Validated delirium-risk assessment, baseline cognitive and functional screening, repeated postoperative delirium surveillance, medication review, adequate analgesia, avoidance of unnecessary deep sedation, early mobility, hydration, sleep support, and timely return of glasses and hearing aids are justified by clinical evidence and consensus recommendations rather than by imaging theory alone (Hughes et al., 2020; Aldecoa et al., 2024). Multicomponent prevention is more consistent with the multifactorial nature of delirium than any single drug or monitor.

The sensory component is practical. Clinicians should document hearing and vision, ensure aids are labeled and available, communicate face-to-face with adequate lighting, reduce avoidable noise at night, provide clear orientation cues, and avoid both deprivation and overload. These actions have favorable risk–benefit profiles even before their neural mediators are established. They should be personalized: a familiar voice may orient one patient, while repeated stimulation may distress another. HELP combines sensory aids, orientation, sleep, and mobility measures (Inouye et al., 1999). Within the proposed model, these components could support input fidelity, contextual updating, and arousal and attention. This remains a mechanistic hypothesis, because clinical benefit from the bundle does not establish mediation through a specific neural pathway.

### 11.2 Anesthetic technique should not be reduced to a binary hierarchy

Randomized comparisons do not support a universal claim that regional or neuraxial anesthesia prevents delirium better than general anesthesia. RAGA found no significant reduction in delirium with regional anesthesia without sedation compared with general anesthesia for hip-fracture surgery (Li et al., 2022). REGAIN likewise found no superiority of spinal over general anesthesia for major functional outcomes and did not establish a broad delirium advantage (Neuman et al., 2021). These trials address clinical strategies, not identical drug-free contrasts; sedation, conversion, surgical context, pain, and postoperative care still matter.

### 11.3 What is plausible but not established

Several interventions are mechanistically attractive: age-adjusted EEG guidance; avoidance of prolonged suppression; individualized blood-pressure management; dexmedetomidine in selected contexts; opioid-sparing analgesia; perioperative sleep and circadian protocols; cognitive or sensory prehabilitation; and post-anesthetic network-targeted stimulation. Trials of cognitive prehabilitation remain promising but heterogeneous (Humeidan et al., 2021), and combined pharmacological regimens such as dexmedetomidine plus acetaminophen require confirmation across procedures and populations (Subramaniam et al., 2019). No intervention should be described as restoring SC–FC coupling until that mediator has been measured prospectively.

## 12 A high-standard research agenda

Figure 5 converts the research agenda into a longitudinal core-versus-enhanced acquisition framework spanning baseline, anesthetic exposure, early recovery, the delirium-risk window, and longer-term cognition.

**Figure 5:**
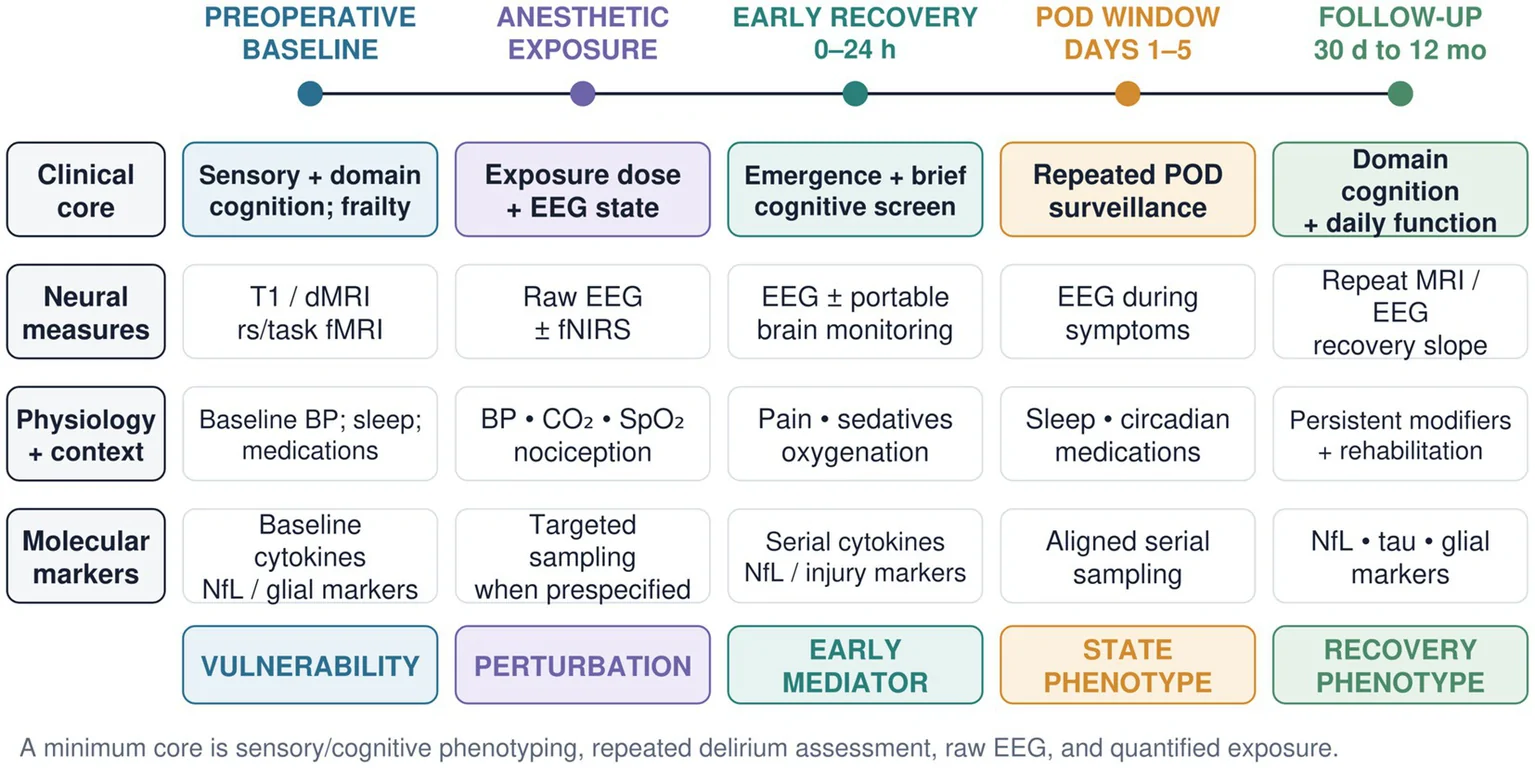
Longitudinal multimodal measurement framework. A minimum clinical core of sensory and cognitive phenotyping, delirium surveillance, raw EEG, and quantified exposure is aligned with enhanced structural MRI, diffusion MRI, resting/task fMRI, perfusion, physiology, and molecular markers. Timing is anchored to baseline, anesthetic exposure, early recovery, the postoperative delirium window, and intermediate or long-term cognitive outcome. The framework separates measures of vulnerability, perturbation, mediator, and phenotype. The days 1–5 sampling window in this illustrative schedule does not define the full POD period. Enhanced measures depend on the study question.

### 12.1 Cohort design and time points

A definitive study should enroll a sufficiently large, representative cohort before major surgery, retain patients with common vulnerability rather than excluding them, and include a non-surgical or minimally exposed comparator when feasible. Imaging and behavioral measurements should be anchored to mechanistically meaningful phases:

- Baseline, sufficiently before premedication to measure reserve, sensory function, cognition, sleep, pain, frailty, mood, and vascular burden;
- Intraoperative, with raw EEG, anesthetic concentrations, analgesia, blood pressure, ventilation, oxygenation, temperature, and major events;
- Early recovery, using bedside EEG and structured delirium assessment when MRI is often infeasible;
- Early postoperative imaging, after documenting residual sedative and physiological state;
- Intermediate recovery at approximately 1–3 months, when delayed recovery and postoperative neurocognitive disorder can be distinguished;
- Longer follow-up at 6–12 months for persistent function, quality of life, and neurodegenerative trajectories.

Repeated measurements before surgery in a subset would quantify test–retest reliability and ordinary temporal variation.

### 12.2 Phenotyping the interface

The clinical battery should combine daily delirium assessment with attention, processing speed, executive control, working memory, and episodic-memory measures. Alternate forms and reliable-change methods should address practice effects.

Sensory phenotyping should include hearing thresholds or speech-in-noise performance, visual acuity and contrast where practical, aid use, pain, interoceptive symptoms, vestibular or proprioceptive problems when relevant, and an audit of the postoperative sensory environment. These variables should be tested as effect modifiers, not only adjusted away as nuisance covariates.

### 12.3 Core multimodal acquisition

A pragmatic MRI core comprises T1-weighted anatomy, FLAIR, diffusion MRI, resting-state fMRI, and arterial spin labeling where feasible. Susceptibility-sensitive and diffusion-weighted sequences can detect microbleeds and acute lesions. A short auditory or multisensory task can probe encoding and feedback, while simultaneous or closely paired EEG links oscillatory routing to macroscale topology. Blood sampling should target prespecified inflammatory and neuronal-injury pathways at aligned times.

Consensus neuroimaging reporting recommendations should guide acquisition and analysis (Nichols et al., 2017). fMRIPrep or an equivalently transparent pipeline can improve reproducibility but does not remove the need to justify denoising choices (Esteban et al., 2019). Scanner and site effects require prospective calibration, phantoms, traveling subjects where feasible, and harmonization that preserves biological variation (Fortin et al., 2018).

### 12.4 Prespecified endpoints and statistical model

Primary endpoints should be few, reliable, and mechanistically ordered. A strong set might include: preoperative segregation across sensory, salience–attention, control, and default-mode systems; thalamocortical gradient geometry; early postoperative EEG feedback or complexity; and the slope of recovery toward an age-appropriate reference. Secondary analyses can examine regional SC–FC coupling, dynamic states, network control, and agent-specific signatures.

Longitudinal mixed-effects or latent-growth models should estimate within-person trajectories. Causal diagrams should be specified before covariate selection because adjusting for post-exposure mediators can create bias. Site, scanner, surgery, and anesthetic strategy require hierarchical modeling rather than simple pooling.

Prediction is a separate objective. Imaging models should be compared with a strong clinical baseline, use nested cross-validation, report calibration and decision-curve utility, and undergo temporal and external validation. Large neuroimaging samples show that small studies can overestimate brain–behavior associations (Marek et al., 2022).

### 12.5 Trials and causal tests

The highest-value trials will randomize a modifiable strategy and measure the proposed neural mediator. Factorial or adaptive designs can separate components, while mediation analysis can test whether improved outcome occurs through the predicted network path.

An anesthetic-strategy trial should not rely on nominal technique labels. It should quantify actual drug concentrations, EEG state, nociceptive and hemodynamic exposure, emergence dynamics, and postoperative care.

### 12.6 Inclusion, feasibility, and missingness

If patients at higher POD risk are less able to tolerate MRI or testing, analyzing only complete cases can introduce selection bias. Protocols should predefine abbreviated bedside assessments, portable EEG or fNIRS, proxy functional outcomes, reasons for missingness, and inverse-probability or joint modeling where appropriate. Scan-completion flow diagrams should compare included and excluded participants by baseline risk and delirium severity. For participants unable to undergo MRI, brief bedside tests of attention and orientation, validated delirium assessment, and portable EEG or fNIRS may allow continued longitudinal follow-up. fNIRS measures superficial cortical hemodynamics and can be affected by systemic physiology (Yücel et al., 2021), so it can supplement but cannot replace whole-brain MRI.

Older adults with sensory impairment, limited education, multiple languages, frailty, or baseline cognitive impairment should not be systematically excluded. Accessible task instructions, sensory correction, culturally appropriate tests, and capacity/consent procedures are scientific necessities, not only ethical accommodations.

## 13 Methodological safeguards and interpretive standards

Table 7 provides a minimum reporting and quality-control checklist for perioperative connectomics.

**Table 7:** Minimum reporting and quality-control checklist for perioperative connectomics.

Domain	Minimum requirement	Why it matters	Report explicitly
Phenotype	Separate POD, delayed recovery, and postoperative NCD; use validated criteria	These outcomes differ in timing and mechanism	Instrument, assessor frequency, severity, subtype, adjudication
Baseline	Obtain preoperative cognition and function	Without baseline, postoperative change cannot be attributed	Battery, timing, education/language adaptation, informant data
Sensory status	Measure hearing and vision; document aids	Sensory loss is a hypothesized modifier and measurement confound	Method, correction, task accessibility, ward availability
Exposure	Quantify delivered anesthetic, analgesia, physiology, and major events	Nominal technique labels conceal actual neural exposure	Dose/concentration, EEG state, BP/CO_2_/O_2_, pain, transfusion, complications
Timing	Anchor scans to drug offset, delirium episode, sleep, and biomarkers	Network state changes rapidly	Exact clock time and interval from each event
Representativeness	Report screening, exclusions, and enrollment	Risk enrichment or exclusion affects generalizability	Flow diagram and reasons by risk group
Missingness	Compare scanned and non-scanned patients; plan bedside alternatives	Sickest patients are least likely to complete MRI	Completion by POD/severity; missing-data model
MRI acquisition	Use harmonized parameters and prospective site QC	Site/scanner differences can dominate subtle effects	Sequence, scanner, coils, phantom/site procedures
Motion	Predefine censoring and sensitivity analyses	Motion correlates with age, pain, cognition, and delirium	Framewise metrics, retained data, group differences, matched-motion result
Physiology	Record respiration/CO_2_, cardiac signal, BP, oxygenation where feasible	BOLD and fNIRS are hemodynamic	Availability, correction model, residual group differences
Arousal/medication	Document eyes, drowsiness, residual sedatives, and analgesics	State and drug effects can mimic network abnormality	Behavioral state, timing, and dose of all CNS-active drugs
Preprocessing	Prespecify transparent pipeline and nuisance strategy	Choices alter correlation structure	Software/version, filtering, global signal, scrubbing, QC
Parcellation	Use a primary atlas and sensitivity atlas; include relevant subcortex/cerebellum	Network boundaries and coupling depend on atlas	Atlas, resolution, transforms, coverage
Diffusion	Report model, correction, tractography, weighting, and edge definition	Structural connectomes are algorithm-dependent	All parameters; absent-edge treatment; lesion handling
SC–FC coupling	Define scale, formula, distance handling, direction, and reference	“Coupling” is not a single metric	Regional/edge/network level; expected sign; normative comparator
Multiplicity	Limit primary outcomes and control family-wise error/FDR	High-dimensional analyses inflate false positives	Confirmatory family, correction, exploratory labeling
Longitudinal model	Model within-person change and informative attrition	Cross-sectional contrasts cannot identify recovery	Random effects, nonlinearity, dropout assumptions
Prediction	Compare with clinical baseline; nested validation; calibration; external test	Internal accuracy can be severely optimistic	Splits, tuning, leakage checks, calibration, decision utility
Causal inference	State causal assumptions; distinguish confounder, mediator, and collider	Overadjustment or selection can reverse effects	DAG or explicit estimand; temporal ordering
Transparency	Preregister; share code, derived features, and QC where permitted	Replication requires inspectable decisions	Registration, repository, deviations, negative results
Interpretation	Avoid “higher is better”; distinguish direct evidence from extrapolation	Connectivity direction is network- and time-specific	Evidence tier, uncertainty, alternatives, falsification criteria

The checklist is intended for protocols, preregistration, reporting, and peer review. ALFF, amplitude of low-frequency fluctuations; APOE4, apolipoprotein E ε4 allele; ASL, arterial spin labeling; BBB, blood–brain barrier; BIS, Bispectral Index; BOLD, blood oxygen level-dependent; BP, blood pressure; CBF, cerebral blood flow; CNS, central nervous system; CO_2_, carbon dioxide; CSF, cerebrospinal fluid; DAG, directed acyclic graph; DAN, dorsal attention network; DCM, dynamic causal modeling; dMRI, diffusion magnetic resonance imaging; DMN, default-mode network; DNR, delayed neurocognitive recovery; DTI, diffusion tensor imaging; DWI, diffusion-weighted imaging; EEG, electroencephalography; FA, fractional anisotropy; FC, functional connectivity; FDR, false discovery rate; FLAIR, fluid-attenuated inversion recovery; fMRI, functional magnetic resonance imaging; fNIRS, functional near-infrared spectroscopy; HbO, oxygenated hemoglobin; HbR, deoxygenated hemoglobin; MD, mean diffusivity; MRI, magnetic resonance imaging; NCD, neurocognitive disorder; NfL, neurofilament light; O2, oxygen; OR, operating room; PMID, PubMed identifier; PND, perioperative neurocognitive disorder; POCD, postoperative cognitive dysfunction (legacy, source-defined term); POD, postoperative delirium; QC, quality control; rs-fMRI, resting-state functional magnetic resonance imaging; SC–FC, structural–functional connectivity; SNc, substantia nigra pars compacta; SNR, signal-to-noise ratio; T1, longitudinal relaxation time (T1-weighted sequence); TMT-B, Trail Making Test Part B; VAN, ventral attention network; VTA, ventral tegmental area; WMH, white-matter hyperintensity.

BALANCED, Balanced Anaesthesia Study; BioCog, Biomarker Development for Postoperative Cognitive Impairment in the Elderly; ENGAGES, Electroencephalography Guidance of Anesthesia to Alleviate Geriatric Syndromes; IPOD-B3, Interventions for Postoperative Delirium: Biomarker-3; RAGA, regional anesthesia versus general anesthesia trial; REGAIN, Regional versus General Anesthesia for Promoting Independence after Hip Fracture; SAGES, Successful Aging after Elective Surgery.

### 13.1 Motion, physiology, and arousal

Head motion can generate distance-dependent connectivity artifacts and correlate with age, cognition, pain, and delirium (Power et al., 2012; Power et al., 2014). Frame censoring, motion models, component-based noise correction, and sensitivity analyses are mandatory, but aggressive denoising can also remove neural signal. Studies should report retained frames, group differences in motion, exclusion thresholds, and whether findings survive matched-motion analyses.

Respiration, carbon dioxide, heart rate, blood pressure, and vascular reactivity affect BOLD signals and can differ systematically after surgery. Physiological recording should be integrated whenever possible. Global-signal regression, scrubbing, and filter choices should be reported because they alter correlation structure and anticorrelations (Ciric et al., 2017). Awake eyes-open or eyes-closed state, drowsiness, residual sedatives, caffeine, analgesics, and time of day should also be documented.

### 13.2 Parcellation, reliability, and multiplicity

Network results depend on parcellation and connectivity-estimation choices (Fotiadis et al., 2024). A primary atlas should be prespecified and key effects tested in at least one alternative parcellation. Reliability should be quantified for the actual scan duration and population. Thousands of edge-wise tests invite selective reporting; network-level hypotheses, permutation testing, false-discovery control, and replication are preferable to unstructured lists of regional findings.

SC–FC analyses require additional transparency: diffusion model and tractography algorithm; streamline weighting; handling of absent edges; transform applied to structural values; functional-connectivity measure; regional versus edge-wise coupling; and treatment of Euclidean distance. Different methods answer different questions. Results should never be summarized as “structure and function became uncoupled” without specifying scale, region, direction, and reference.

### 13.3 Neurovascular and pharmacological interpretation

Vascular physiology can influence BOLD connectivity (Golestani et al., 2016). We recommend multimodal EEG–fMRI, perfusion measures, physiological covariates, and agent-specific controls to assess the contribution of vascular effects. Postoperative scans should record all centrally active medications and their timing. A correlation acquired during active delirium cannot be assumed to represent the same mechanism as a preoperative predictor or a three-month residual change.

### 13.4 Transparent reporting and reproducibility

Preregistration should distinguish confirmatory from exploratory endpoints. Acquisition parameters, preprocessing code, quality-control criteria, parcellations, and statistical models should be shared where consent and governance permit. Site-stratified results and uncertainty intervals matter more than a single pooled *p* value.

## 14 Discussion

### 14.1 What the framework adds

The framework proposes explanations for why early sensory responses can be preserved despite impaired cognitive access, why both unimodal and association systems are involved in perioperative studies, and why recovery topology and rate may be more informative than the magnitude of acute suppression. Its specific hypotheses distinguish early sensory encoding from the subsequent use of that information in context, and test whether sensory reserve modifies POD-related network change. Vulnerability, disconnection, and failed recovery are established concepts. Whether these additional measurements improve the explanation of POD remains unproven.

### 14.2 Boundary conditions and competing explanations

Global slowing, whole-brain connectivity loss, or elevated cytokines alone do not uniquely support a sensory–cognitive account. The model earns explanatory value only if sensory measures and hierarchical network metrics add reproducible information beyond age, frailty, baseline cognition, conventional MRI burden, achieved anesthetic exposure, and perioperative physiology. Competing accounts include strategic vascular injury, diffuse neuroimmune or neuromodulatory failure without privileged sensory involvement, residual drug effect, and latent neurodegeneration unmasked by surgery. These mechanisms are not mutually exclusive and may define biologically distinct subgroups. A useful future analysis should therefore compare models rather than enter all candidate variables into one undifferentiated predictor set.

### 14.3 Limitations of the evidence and this synthesis

Direct sensory-specific perioperative evidence is still narrow: the most compelling observations come from auditory prediction-error EEG and a small number of visual-network studies (Gjini et al., 2024; Zhang G. et al., 2024), whereas much of the proposed hierarchy is extrapolated from anesthesia, consciousness, aging, or non-surgical neuroscience. Perioperative cohorts are generally modest, phenotype definitions and imaging time points vary, active-delirium MRI is rare, postoperative attrition is informative, and repeated analyses of the same cohorts can create an impression of replication. Functional-connectivity direction is inconsistent across studies, and BOLD findings remain vulnerable to motion, arousal, medication, carbon dioxide, and neurovascular effects. Anesthesia-state evidence often derives from younger volunteers and controlled exposures that do not reproduce the older surgical brain. Animal models clarify immune pathways but cannot reproduce the human cognitive phenotype.

This critical narrative synthesis was not protocol-registered and did not use duplicate screening, formal risk-of-bias scoring, or meta-analysis. Selection and interpretive bias therefore remain possible despite deliberate inclusion of null and discordant findings. These hypotheses need prospective testing to distinguish the proposed mechanisms. The review does not validate an imaging biomarker or support routine clinical fMRI.

## 15 Conclusion

POD is unlikely to arise from a single region, molecule, or anesthetic exposure. The sensory–cognitive interface offers a proposed systems-level account of how afferent information is relayed, integrated, weighted, and used in context to support attention, memory, and executive control.

Direct human studies demonstrate preoperative dysconnectivity, altered sensory and association-network organization, abnormal slow-wave and feedback dynamics, and incomplete recovery after POD (Tanabe et al., 2020; Ditzel et al., 2023; Gjini et al., 2024; Taylor et al., 2026), but they do not establish one causal pathway. Support for the framework requires replicated, out-of-sample evidence that sensory phenotypes, hierarchical metrics, and within-person trajectories add value beyond conventional vulnerability and competing mechanisms. It also requires evidence that an intervention changes both the predicted mediator and cognition. Otherwise, the framework should be narrowed. Extension to delayed neurocognitive recovery or postoperative neurocognitive disorder requires separate outcome-specific evidence.
