# Supplementary material for: The perioperative sensory–cognitive interface: multimodal neuroimaging of hierarchical network reconfiguration in perioperative neurocognitive disorders
Source: Front Neurosci. 2026 Oct 21;20:1956385. doi: 10.3389/fnins.2026.1956385 (PMC13760687; doi:10.3389/fnins.2026.1956385)
Supplement: Supplementary file 1 [file Supplementary_file_1.docx]

Supplementary Methods

# Targeted literature search supporting the narrative review

Five targeted search modules were executed in PubMed and Europe PMC. They addressed postoperative delirium (POD) in relation to sensory processing and electroencephalography (EEG); MRI and brain-network measures; human intracranial evidence concerning anesthetic thalamocortical effects; ketamine or esketamine in older surgical patients; and EEG-guided anesthetic depth.

The review focuses on POD. Delayed and longer-term postoperative cognitive outcomes are considered separately to examine recovery and the limits of the proposed framework. Studies were selected according to their relevance to the review question. This was a narrative review, not a systematic review update, and the selection principles below were not prospectively registered eligibility criteria.

# Databases, dates, and retrieval

PubMed (https://pubmed.ncbi.nlm.nih.gov/) was queried through NCBI E-utilities ESearch, with JSON output, a maximum of 10,000 returned identifiers per query, and sorting by publication date. All returned identifiers were retrieved. The exact strings used title/abstract fields and a publication-date interval of 1 January 1800 to 21 September 2026.

Europe PMC (https://europepmc.org/) was queried through its REST search interface, with JSON lightweight records and a page size of 1,000. All returned records were retrieved. Searches used title/abstract fields, were restricted to MED records (SRC:MED), and used the FIRST_PDATE interval of 1 January 1800 to 21 September 2026.

No language filter was applied. No universal human-study or randomized-trial filter was imposed beyond the terms explicitly included in each string. Population, study design, outcome definition, and conceptual relevance were considered during narrative selection. An article published electronically within the specified interval can appear under a later print-issue date. Database-specific date fields and indexing also contribute to differences in returned counts.

# Database returns

| **Search module** | **PubMed identifiers returned** | **Europe PMC records returned** |
| --- | --- | --- |
| Sensory processing / EEG and POD | 576 | 535 |
| MRI / network measures and POD | 102 | 88 |
| Human intracranial anesthetic thalamocortical evidence | 44 | 39 |
| Ketamine / esketamine and POD in older adults | 77 | 72 |
| EEG-guided depth / BALANCED / ENGAGES and delirium trials | 110 | 103 |

These numbers are database hit counts, not the numbers of studies screened or included. Because queries and databases overlap, adding the counts would not give the number of unique or independent studies. The counts do not provide a denominator for an exhaustive review or a PRISMA study flow.

# Selection principles for Table 4

We prioritized original human studies that linked perioperative MRI or EEG measurements to a defined delirium or cognitive outcome. Selected studies examined baseline vulnerability, active POD, or postoperative recovery. Measurement timing was stated because these settings have different implications for causal interpretation.

We retained informative null and discordant findings alongside positive associations. Selection did not depend solely on statistical significance or agreement with the proposed framework. Publications drawing on the same parent cohort were identified where this was apparent, so the number of publications does not equal the number of independent cohorts.

POD was distinguished from delayed and longer-term cognitive change. Findings on later cognitive change were used to examine the scope and limitations of the framework without assuming a shared mechanism. Animal experiments, healthy-volunteer anesthesia studies, protocols, reviews, pediatric emergence-delirium studies, and purely behavioral intervention trials were not classified as direct MRI/EEG evidence of POD mechanisms. Where relevant, they were discussed in separately labeled sections.

Connectivity changes were not taken as evidence specific to the sensory–cognitive interface unless they could be related explicitly to sensory processing, routing or contextualization, and behavioral interpretation. Human anesthesia-state studies, aging research, and methodological studies were considered supporting evidence from related fields, with limits on the conclusions that could be drawn for perioperative patients.

These principles describe how studies were selected for the revised narrative review; they were not prespecified screening rules. We did not screen all retrieved records, use two independent reviewers for selection, formally score study-level risk of bias, or conduct a meta-analysis. The review may therefore omit relevant studies and remains subject to selection bias.

# Exact database search strings

Exact search strings are listed below. Counts were obtained on 21 September 2026 and may change as records are indexed or corrected.

## P1 — PubMed: POD, sensory processing, and EEG

("postoperative delirium"[Title/Abstract] OR "post-operative delirium"[Title/Abstract] OR (delirium[Title/Abstract] AND (perioperative[Title/Abstract] OR postsurgical[Title/Abstract]))) AND (sensory[Title/Abstract] OR auditory[Title/Abstract] OR "mismatch negativity"[Title/Abstract] OR EEG[Title/Abstract] OR electroencephalogra*[Title/Abstract] OR hearing[Title/Abstract] OR visual[Title/Abstract]) AND ("1800/01/01"[Date - Publication] : "2026/09/21"[Date - Publication])

Returned count: 576; retrieved identifiers: 576.

## P2 — PubMed: POD, MRI, and brain networks

("postoperative delirium"[Title/Abstract] OR "post-operative delirium"[Title/Abstract] OR (delirium[Title/Abstract] AND (perioperative[Title/Abstract] OR postsurgical[Title/Abstract]))) AND (MRI[Title/Abstract] OR fMRI[Title/Abstract] OR "functional connectivity"[Title/Abstract] OR "structural connectivity"[Title/Abstract] OR "brain network"[Title/Abstract] OR neuroimag*[Title/Abstract] OR "structure-function"[Title/Abstract]) AND ("1800/01/01"[Date - Publication] : "2026/09/21"[Date - Publication])

Returned count: 102; retrieved identifiers: 102.

## P3 — PubMed: Human intracranial anesthetic and thalamocortical evidence

(anesthe*[Title/Abstract] OR anaesthe*[Title/Abstract] OR propofol[Title/Abstract]) AND (thalam*[Title/Abstract] OR thalamocortical[Title/Abstract]) AND (intracranial[Title/Abstract] OR electrocorticogra*[Title/Abstract] OR "local field potential"[Title/Abstract]) AND (human*[Title/Abstract] OR patient*[Title/Abstract]) AND ("1800/01/01"[Date - Publication] : "2026/09/21"[Date - Publication])

Returned count: 44; retrieved identifiers: 44.

## P4 — PubMed: Ketamine and postoperative delirium in older adults

("postoperative delirium"[Title/Abstract] OR "post-operative delirium"[Title/Abstract] OR (delirium[Title/Abstract] AND (perioperative[Title/Abstract] OR postsurgical[Title/Abstract]))) AND (ketamine[Title/Abstract] OR esketamine[Title/Abstract]) AND (older[Title/Abstract] OR elderly[Title/Abstract] OR aged[Title/Abstract] OR geriatric[Title/Abstract]) AND ("1800/01/01"[Date - Publication] : "2026/09/21"[Date - Publication])

Returned count: 77; retrieved identifiers: 77.

## P5 — PubMed: EEG-guided depth, BALANCED, and delirium trials

("postoperative delirium"[Title/Abstract] OR "post-operative delirium"[Title/Abstract] OR (delirium[Title/Abstract] AND (perioperative[Title/Abstract] OR postsurgical[Title/Abstract]))) AND (BALANCED[Title/Abstract] OR ENGAGES[Title/Abstract] OR bispectral[Title/Abstract] OR "anaesthetic depth"[Title/Abstract] OR "anesthetic depth"[Title/Abstract] OR "EEG-guided"[Title/Abstract] OR "electroencephalography-guided"[Title/Abstract]) AND (random*[Title/Abstract] OR trial[Title/Abstract]) AND ("1800/01/01"[Date - Publication] : "2026/09/21"[Date - Publication])

Returned count: 110; retrieved identifiers: 110.

## S1 — Europe PMC: POD, sensory processing, and EEG

(TITLE_ABS:"postoperative delirium" OR TITLE_ABS:"post-operative delirium" OR (TITLE_ABS:delirium AND (TITLE_ABS:perioperative OR TITLE_ABS:postsurgical))) AND (TITLE_ABS:sensory OR TITLE_ABS:auditory OR TITLE_ABS:"mismatch negativity" OR TITLE_ABS:EEG OR TITLE_ABS:electroencephalogra* OR TITLE_ABS:hearing OR TITLE_ABS:visual) AND SRC:MED AND FIRST_PDATE:[1800-01-01 TO 2026-09-21]

Returned count: 535; retrieved records: 535.

## S2 — Europe PMC: POD, MRI, and brain networks

(TITLE_ABS:"postoperative delirium" OR TITLE_ABS:"post-operative delirium" OR (TITLE_ABS:delirium AND (TITLE_ABS:perioperative OR TITLE_ABS:postsurgical))) AND (TITLE_ABS:MRI OR TITLE_ABS:fMRI OR TITLE_ABS:"functional connectivity" OR TITLE_ABS:"structural connectivity" OR TITLE_ABS:"brain network" OR TITLE_ABS:neuroimag* OR TITLE_ABS:"structure-function") AND SRC:MED AND FIRST_PDATE:[1800-01-01 TO 2026-09-21]

Returned count: 88; retrieved records: 88.

## S3 — Europe PMC: Human intracranial anesthetic and thalamocortical evidence

(TITLE_ABS:anesthe* OR TITLE_ABS:anaesthe* OR TITLE_ABS:propofol) AND (TITLE_ABS:thalam* OR TITLE_ABS:thalamocortical) AND (TITLE_ABS:intracranial OR TITLE_ABS:electrocorticogra* OR TITLE_ABS:"local field potential") AND (TITLE_ABS:human* OR TITLE_ABS:patient*) AND SRC:MED AND FIRST_PDATE:[1800-01-01 TO 2026-09-21]

Returned count: 39; retrieved records: 39.

## S4 — Europe PMC: Ketamine and postoperative delirium in older adults

(TITLE_ABS:"postoperative delirium" OR TITLE_ABS:"post-operative delirium" OR (TITLE_ABS:delirium AND (TITLE_ABS:perioperative OR TITLE_ABS:postsurgical))) AND (TITLE_ABS:ketamine OR TITLE_ABS:esketamine) AND (TITLE_ABS:older OR TITLE_ABS:elderly OR TITLE_ABS:aged OR TITLE_ABS:geriatric) AND SRC:MED AND FIRST_PDATE:[1800-01-01 TO 2026-09-21]

Returned count: 72; retrieved records: 72.

## S5 — Europe PMC: EEG-guided depth, BALANCED, and delirium trials

(TITLE_ABS:"postoperative delirium" OR TITLE_ABS:"post-operative delirium" OR (TITLE_ABS:delirium AND (TITLE_ABS:perioperative OR TITLE_ABS:postsurgical))) AND (TITLE_ABS:BALANCED OR TITLE_ABS:ENGAGES OR TITLE_ABS:bispectral OR TITLE_ABS:"anaesthetic depth" OR TITLE_ABS:"anesthetic depth" OR TITLE_ABS:"EEG-guided" OR TITLE_ABS:"electroencephalography-guided") AND (TITLE_ABS:random* OR TITLE_ABS:trial) AND SRC:MED AND FIRST_PDATE:[1800-01-01 TO 2026-09-21]

Returned count: 103; retrieved records: 103.
